# DomPep—A General Method for Predicting Modular Domain-Mediated Protein-Protein Interactions

**DOI:** 10.1371/journal.pone.0025528

**Published:** 2011-10-07

**Authors:** Lei Li, Bing Zhao, Jun Du, Kaizhong Zhang, Charles X. Ling, Shawn Shun-Cheng Li

**Affiliations:** 1 Department of Biochemistry, Siebens-Drake Medical Research Institute, Schulich School of Medicine and Dentistry, The University of Western Ontario, London, Ontario, Canada; 2 Department of Computer Science, The University of Western Ontario, London, Ontario, Canada; Instituto Nacional de Câncer, Brazil

## Abstract

Protein-protein interactions (PPIs) are frequently mediated by the binding of a modular domain in one protein to a short, linear peptide motif in its partner. The advent of proteomic methods such as peptide and protein arrays has led to the accumulation of a wealth of interaction data for modular interaction domains. Although several computational programs have been developed to predict modular domain-mediated PPI events, they are often restricted to a given domain type. We describe DomPep, a method that can potentially be used to predict PPIs mediated by any modular domains. DomPep combines proteomic data with sequence information to achieve high accuracy and high coverage in PPI prediction. Proteomic binding data were employed to determine a simple yet novel parameter Ligand-Binding Similarity which, in turn, is used to calibrate Domain Sequence Identity and Position-Weighted-Matrix distance, two parameters that are used in constructing prediction models. Moreover, DomPep can be used to predict PPIs for both domains with experimental binding data and those without. Using the PDZ and SH2 domain families as test cases, we show that DomPep can predict PPIs with accuracies superior to existing methods. To evaluate DomPep as a discovery tool, we deployed DomPep to identify interactions mediated by three human PDZ domains. Subsequent in-solution binding assays validated the high accuracy of DomPep in predicting authentic PPIs at the proteome scale. Because DomPep makes use of only interaction data and the primary sequence of a domain, it can be readily expanded to include other types of modular domains.

## Introduction

Cellular signal transduction is regulated by the formation and dissolution of specific protein complexes. These complexes is often mediated by modular protein-interaction domains such as the Src homology 2 (SH2) or 3 (SH3) and the Post-synaptic density protein, Disc large, Zonula occludens-1 (or PDZ) domain that are capable of binding partner proteins via short peptide motifs [1]. While distinct families of modular domains differ in the three-dimensional fold and ligand-binding specificity [2], [3], members of the same family are related by the same fold and similar specificity [4], [5]. Both extrinsic and intrinsic factors are thought to contribute to the specificity of a modular domain. Extrinsic factors, which include sub-cellular localization and contextual information [6], [7], are difficult to predict *in silico*. Intrinsic specificity determinants, such as the amino acid sequence of a domain and the characteristics of its ligand-binding site, are context-independent because they are retained even in the isolated domains [8]. In fact, much of our current knowledge on modular domain-ligand recognition has been gleaned from studies using isolated domains and short peptides [9], [10], [11]. For example, synthetic peptide libraries and oriented peptide array libraries were used to gauge the specificity of the SH2 domain [12] whereas phage-displayed peptide libraries were employed to determine the specificity of the PDZ and SH3 domains [13]. Recently, peptide and domain arrays were employed for large-scale PPI identification [14], [15]. The resulting specificity data are invaluable not only for rationalizing known interactions but for constructing *in silico* models to predict novel PPI events [16], [17].

Intrinsic specificity information of a domain, obtained from sequence analysis and/or experimental data on domain-peptide binding, may be harnessed for *in silico* prediction of binding partners for that domain or for other members of the same domain family. Scansite [18], NetPhorest [16] and SMALI [19] are but three examples of the many approaches developed for prediction of PPI events mediated by modular domains. The performance of a given method depends on the quality of the prediction models, which, in turn, is dictated by the quality and quantity of the relevant experimental binding data. While sufficient experimental binding data may be available for certain domains to build the corresponding prediction models or classifiers, it may be scarce or completely missing for other domains. To overcome this limitation imposed by lack of experimental data, domains that share similar specificities are often grouped together such that their binding ligands may be pooled together to create a common classifier [16], [20]. A common classifier usually leads to improved predictive performance because the number of positives and negatives used in the training increases significantly when experimental binding data from multiple domains are combined. Despite this apparent advantage, currently there is no general standard that can be used to identify domains with similar specificities. While domain sequence identity (DSI) provides a good measure of the evolutionary distance between two domains, it is complicated by the different threshold values used in different studies [21], [22], [23]. To overcome this potential problem, Xu *et al.* [20] and Miller *et al.* [16] developed methods to classify and group domains based on the phylogenic tree of the domain family. In this scheme, domains from the same branch of the phylogenetic tree are considered similar in specificity and therefore grouped together. Nevertheless, DSI values differ widely from one branch to another, making it difficult to define a cut-off value by which to discern whether two domains belonging to the same branch of the phylogenic tree have similar specificity or not. For example, members of Src family SH2 domains have greater than 50% DSI and form a branch in the SH2 domain phylogenetic tree. In contrast, the SOCS family SH2 domains, which forms another branch, share ∼25% DSI [24]. These limitations suggest that it is necessary to develop a new strategy that integrates specificity information with sequence identity to classify and group domains with related binding properties.

While the above methods make use of specificity and sequence information for PPI prediction, other approaches exploit the binding site characteristics of the relevant domain-peptide complex structures. For instance, a position-specific domain-ligand-contact frequency matrix could be constructed from the contacting residues in the corresponding domain-peptide complex structures [25], [26]. Machine learning algorithms, such as support vector machine (SVM), have been developed using the same structural information [27], [28]. While a structure-based predictive algorithm usually performs well for a domain whose binding pocket aligns well with the template structure(s), its application is limited by the quality of the template structure and by the accuracy of structure-based sequence alignment. Moreover, the performance of these structure-based predictive methods depends on the amount of interaction data available for training because the number of potential combinations of contacting residues between the domain and the peptide may exceed the number of data points available for training [25].

To overcome limitations of the existing methods for domain-based PPI prediction, we developed DomPep. Unlike previous methods that group related domains according to a single parameter (e.g., DSI or branches in a phylogenetic tree), DomPep identifies specificity-similar domains by combining two parameters, namely DSI and Position-Weighted-Matrix (PWM) distance. PWM, represented by the sequence logo of the bound peptides [29], provides a measure of domain specificity based on experimental binding data. PWM distance has been employed to measure the difference in binding specificity between two domains, independently of DSI [13]. Although PWM distance and DSI, when used together, provide valuable information on the relatedness of domain specificity, the corresponding threshold values have to be defined. We therefore created a novel parameter called ligand-binding similarity (LBS) by which to calibrate the threshold values for DSI and PWM distance. The LBS of two domains is positively correlated with the number of peptides from the same pool that are recognized by both domains.

Using this new strategy of domain classification, we have built DomPep prediction models for the SH2 and PDZ domains that differ significantly in specificity. The accuracy of prediction in each case was benchmarked against experimental data [13], [16], [20], [21]. The performance of DomPep was compared to that of NetPhorest using its artificial neural network (ANN) models [16] to identify SH2-binding peptides/proteins. In the case of predicting PDZ-ligand interactions, we evaluated DomPep against both SPSSM (Structure-based PSSM) [25] and MDSM (Multi-Domain Selectivity Model) [15] algorithms. DomPep compared favorably to these existing methods when independent training and test sets were used. Moreover, we used DomPep to predict, in silico, ligands for the Scrib PDZ domains from the human protein database. Subsequent binding assays on 56 candidate PDZ-binding ligands indicated that DomPep was effective in distinguishing positive from negative binders. The current online DomPep server could be used to predict ligands for 174 PDZ domains and 97 SH2 domains from different species. We expect DomPep to function as a bioinformatic platform for comparison of domain specificity and as a discovery tool for in silico prediction of domain-peptide interactions and the corresponding PPI networks.

## Results

### Definitions and calculations of PWM distance and LBS

DomPep employs both the DSI and PWM distance to assess the similarity in specificity for two domains. The PWM of a domain is a matrix (such as that represented by a sequence LOGO, [29]) generated by aligning all experimentally determined peptide ligands for that domain. The PWM distance is defined as the difference in PWMs for two domains. Since the PWM distance is based on peptide binding data whereas DSI is calculated from sequence information, they are complementary to each other in identifying domain pairs with similar specificities. Nevertheless, a critical question in applying these two parameters to identify specificity-similar domains is how to set the corresponding threshold values. To address this question, we introduced LBS to provide an empirical measure of the relative specificity of two domains by taking advantage of high-throughput domain-peptide array data. We reasoned that, if the two domains are capable of binding to the same set of peptides, they should possess the same specificity. Therefore, we define LBS as the ratio of the number of peptides commonly recognized by two domains over the total number of peptides that have been experimentally tested (see methods for details). We define two domains to have identical specificity if LBS = 1. Conversely, a LBS value of 0 implies that the specificity of the two domains under concern is non-overlapping. Since the accuracy of the LBS and PWM distance values is determined by the number of binding peptides, we calculated these two parameters for a domain only when it has at least 10 experimentally identified binding peptides. We considered two domains to have similar specificities when LBS ≥ 0.7 as this value was a reasonable compromise between domain coverage and stringency of prediction. This LBS value (i.e. 0.7) was used to calculate the threshold values for DSI and PWM distance, respectively. The latter parameters were then used to identify domains with similar specificity based on the available binding data, as outlined below. It should be emphasized, that due to the limited size of available data on domain-peptide interaction, it is not possible to build individual prediction models for all domains. For a domain with only a few identified peptide ligands, a model may be built by combining its binding data with those of specificity-similar domains. Moreover, the accuracy of a model may be enhanced by an increase in the training peptide set that combine all related binding data.

### The general scheme of DomPep

**Figure 1:**
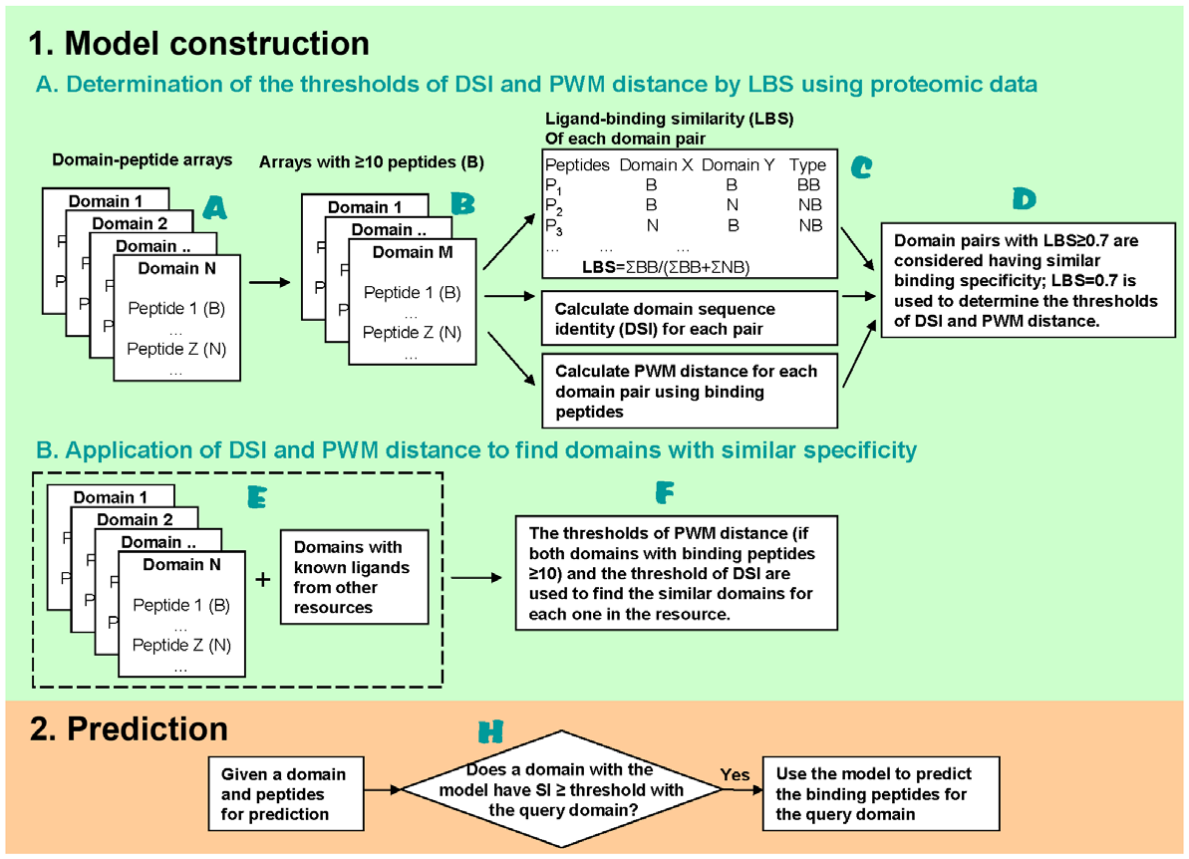
A schematic representation of the DomPep methodology.

DomPep comprises two modules – model construction and PPI prediction (Fig. 1). The model construction module includes two parts: (i) determination of the thresholds of DSI and PWM distance by LBS based on available domain-peptide binding data (Fig.1A–D); (ii) identification and grouping of specificity-similar domains using DSI and PWM distance (Fig. 1E–G). For the first part, domains with a minimum of ten experimentally (ie. domain-peptide array binding) verified peptide ligands are retained for parameter calculations (Fig. 1A &B). Specifically, LBS, PWM distance and DSI values are calculated for all domain pairs (Fig. 1C). A LBS value of 0.7 is set as the cut-off to determine the threshold values of DSI and PWM distance (Fig. 1D). All available experimental binding data (ie. both *in vitro* or *in vivo*) are used to calculate the PWM distance (Fig.1E & F). Domain pairs with PWM distance and DSI values above the corresponding thresholds are considered to have similar specificity. It should be noted that the integration of in vivo and in vitro data for the identification of specificity-similar domains would minimize the effect on predictive performance of non-specific binding events (i.e. false positives) that can potentially originate from in vitro array screening experiments. Based on available experimental binding data (*in vitro* and *in vivo*), the specificity-similar domains to a query domain are identified. These domains are iteratively grouped together with the query and the binding data for each group of the domains are employed to construct prediction models. These models are compared, using leave-one-out cross-validation, with the one constructed using the peptides of the query itself and the model with the best accuracy is retained as the final model for the query. The same procedure is repeated for every domain for model construction. We employed the SVM algorithm to build the DomPep models [27], [28]. The combined use of the three related parameters, LBS, DSI and PWM distance, makes it possible to apply DomPep not only to domains with experimentally derived models but also to those without any experimental binding data. In the latter case, the sequence of the query domain is used to search for a “similar” domain in the family that has a DSI value above the threshold (Fig. 1H). The model for the latter domain is then used for PPI prediction for the query domain.

The utility of the DomPep was demonstrated on the PDZ and SH2 domain families that have distinct specificities and for which comprehensive binding data are available from large-scale proteomic analysis and conventional experiments [13], [16], [25], [30]. We evaluated DomPep through direct comparisons with three recently developed prediction programs and by a genome-scale prediction of PDZ binding ligands followed by experimental validation. Results from these studies are described below.

### Application of DomPep to the PDZ domain family

We analyzed the specificity of PDZ domains based on published data. MacBeath and colleagues recently conducted a study to characterize the specificity of 157 mouse PDZ domains using 217 peptides representing potential physiological PDZ-binding sites [15]. Interactions identified from the PDZ protein array screening were further analyzed by quantitative fluorescence polarization. When a K_d_ cutoff of 100 µM was applied, 731 positive interactions and 1,361 negative interactions were identified that involved 85 PDZ domains and 181 peptides [15]. This set of quantitative binding data was directly employed in our analysis and the same K_d_ cutoff was adopted. The five C-terminal residues of the bound peptides were considered in our analysis as they were shown to be the major specificity-determinants in PDZ binding [13] and were found to provide a good balance between false-positive and false-negative predictions in previous studies [15].

**Figure 2:**
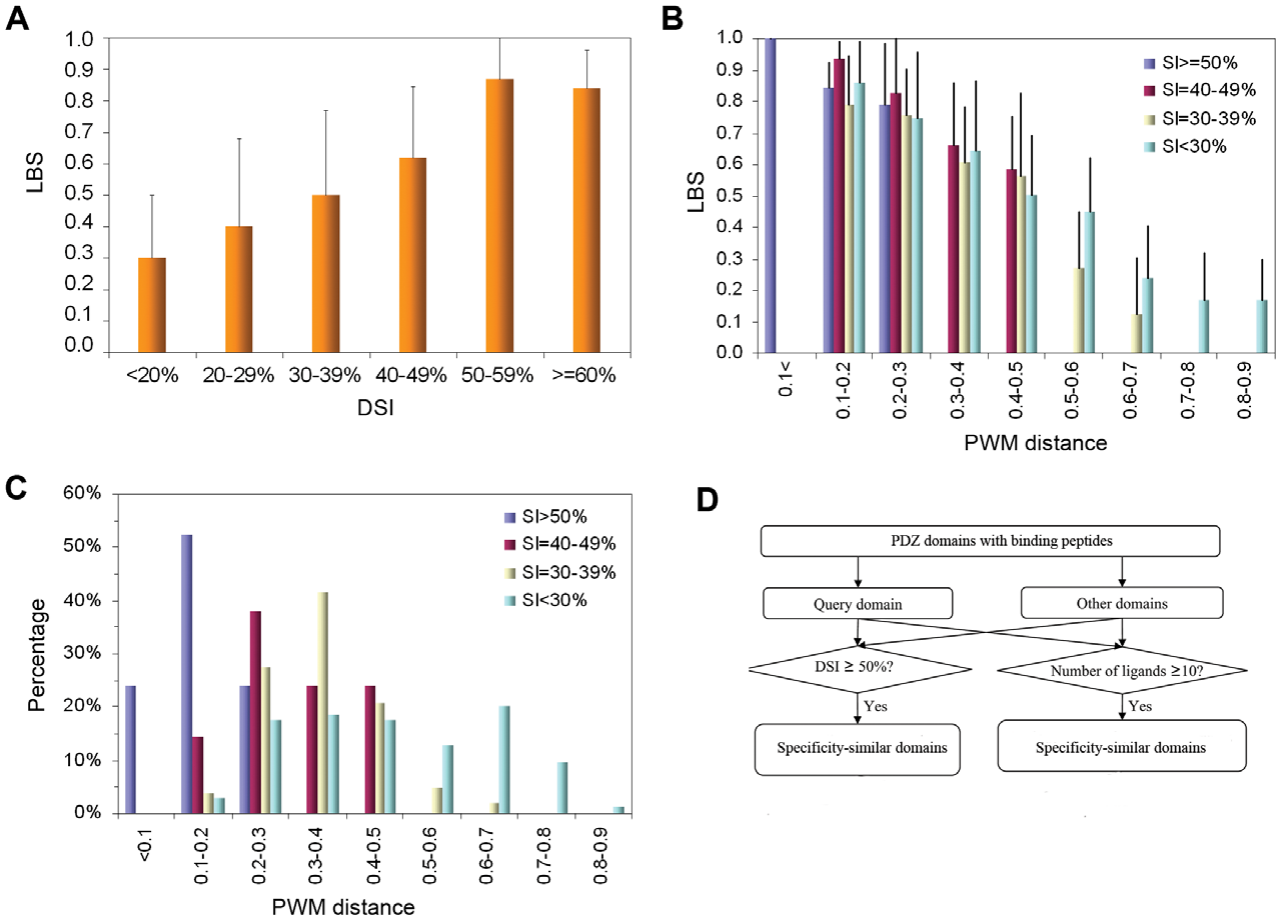
Correlation of LBS, DSI and PWM distance with each other and with the specificity of the PDZ domain. (A) The relationship between ligand binding similarity (LBS) and domain sequence identity (DSI) for PDZ domain pairs based on domain-peptide array binding data. DSI values were calculated according to the ClustalW2 program [45]. (B) The relationship between PWM distance and LBS for PDZ domain pairs in different DSI groups. LBS appears more closely related to PWM distance than to DSI. (C) The relationship of PWM distance to DSI. The percentage of PDZ pairs within a given DSI group was plotted against the corresponding PWM distance. (D) A strategy for the identification of specificity-similar domains for a PDZ domain. This strategy takes advantage of results generated from (A) and (B).

We first investigated the relationship between LBS and DSI. To ensure that the calculation for LBS and subsequently, for PWM distance was accurate, PDZ domains with fewer than 10 experimentally verified ligands were omitted (Fig. 1B). As shown in Figure 2A, PDZ pairs with DSI≥50% had an average LBS value of 0.85. No significant difference in LBS was observed between pairs with DSI = 50-59% or those with DSI>60%. This suggests that domain pairs with sequence identity greater than 50% share the same pool of ligands and are therefore similar in specificity. In contrast, domain pairs with DSI<30% had the smallest average LBS (0.40), suggesting that domains with diverse sequences have distinct specificity. These analyses demonstrate that DSI and LBS are correlated in such a way that the greater the domain sequence identity, the greater the similarity in ligand-binding. While this observation is in agreement with previous findings [13], the correlation deteriorated rapidly with decreasing DSI value. This suggests that, for a model constructed based on DSI, caution should be taken with regard to the minimum DSI value at which homology modeling may be applied. Because PDZ domains with different specificity may still share a certain number of binding peptides, it is not suitable to include the peptides that bind to one PDZ domain in the negative training set of peptides for other PDZ domains. Therefore, we generated the negative training set by randomly selection of peptides from the Swiss-Prot protein database when constructing DomPep models (see Methods for details).

To further explore the relationship between DSI and binding specificity, we calculated the PWM distances (see Methods). PWM distances range from 0 to 1, in which “0” suggests identical specificity and “1” suggests completely different specificity. We graphed LBS against PWM distance and grouped together pairs of domains belonging to the same DSI group (Fig. 2B). As expected, LBS generally decreased with an increase in PWM distance. However, this reverse correlation between LBS and PWM distance, two parameters that measure domain specificity, was seen to be largely independent of DSI. For instance, the pairs with PWM distance of 0.1 to 0.2 have similar LBS values (ie. 0.85) although the corresponding DSI values range from <30% to >50%. Additionally, some PDZ domains display low DSI but high LBS and small PWM distance. For example, the second PDZ domain of DLG3 [or DLG3(2/3)] shares 17% sequence identity with the second PDZ domain of MAGI3 [or MAGI3(2/5)], yet they have a short PWM distance ( = 0.16) and a large LBS ( = 0.94) because they share 15 of 16 peptide ligands tested. This phenomenon is also observed on data obtained using phage-displayed random peptide libraries [13]. For instance, the MAGI3(4/6) and MAGI1(5/6) PDZ domains were found to have similar specificity and therefore classified into the same group [31] despite of a low DSI (24%). These two domains showed a small PWM distance of 0.22 in our analysis (For more examples, see Figure S1).

To characterize the relationship between domain sequence identity and ligand-binding specificity, we plotted the percentile distribution of different DSI groups against the PWM distance. As shown in Fig. 2C, while PDZ pairs with high DSI values generally exhibit small PWM distances, the reverse is not true. With decreases in the DSI, the variations in the PWM distance become wide. However, many domain pairs that share low sequence identity still exhibit small PWM distance values. For example, 28% of PDZ domains in the SI = 30-39% group had PWM distance of 0.2-0.3. Even for the most sequence-diverse group (i.e. DSI<30%), 17% of PDZ pairs have PWM distances within this range. These data indicate that low sequence identity between two PDZ domains does not necessarily imply distinct specificities [13], [15].

PDZ domains with 10 or more binding peptides identified from the arrays were analyzed in pairs to generate the corresponding DSI, PWM distance and LBS values. We used the corresponding LBS value to calibrate DSI and PWM distances. To this end, we arbitrarily set LBS = 0.7 as the cutoff to assign two PDZ domains similar (when LBS ≥ 0.7) or dissimilar (when LBS<0.7). This cutoff value was used, in turn, to determine the threshold values for DSI and PWM distance (Fig. 1D). In the case of the PDZ domains, LBS≥0.7 corresponds to DSI≥50% (Fig. 2A) or PWM distance <0.3 (Fig. 2B). These cutoff/threshold values were subsequently applied to the PDZ domain family to identify domain pairs with similar specificity (Fig. 2D). Specifically, a PDZ domain was sequence-aligned to another domain in the PDZ family and the corresponding DSI value was calculated. If DSI ≥ 50%, the corresponding pair of domains was considered similar in specificity. If the query domain had more than 10 experimentally verified binding peptides, the PWM distance was used to identify another domain that also had at least 10 known ligands. Two domains of this category that have the PWM distance <0.3 were considered specificity-similar. The specificity-similar domains (based on DSI and/or PWM distance) were iteratively grouped with the query domain and their peptides were pooled to form the positive training set for prediction models. The negative training peptides were chosen randomly from the C-termini of proteins in the Swiss-Prot database [15], [25]. The negative training set was, in general, five times greater in number than the positive set [16]. The prediction models for these groups were compared, based on leave-one-out cross-validation, with the model of the query that was constructed using the peptide ligands of the query itself. The model that has the best performance is retained as the final prediction model for the query. For a PDZ domain without a prediction model, we performed the prediction using the model of a related domain with DSI >50%.

### Comparison of DomPep with other methods for predicting PDZ domain-ligand interactions

We compared DomPep to two methods developed recently by MacBeath and coworkers [15], [25] for accuracy in predicting PDZ-ligand interactions. The first method, multi-domain selectivity model or MDSM is a variation of PSSM [15]. In contrast to PSSM that measures the weighted contribution of a residue to binding at a given position of the ligand, MDSM captures the difference in selectivity for different PDZ domains [15]. The second method, called SPSSM, contains structure-based position-specific scoring matrices derived from 38 pairs of interacting residues in a reference PDZ domain-peptide complex structure [25]. While the MDSM models could be used to predict binding ligands for 74 mouse PDZ domains, the SPSSM model is capable, in principle, of predicting interactions for any PDZ domain.

The training set was previously employed to construct both the MDSM and SPSSM models [15], [25].Based on this training set, 66 DomPep models and 74 MDSM models were constructed, of which 54 were common for both methods. The independent test set was composed of 48 mouse PDZ domain-containing proteins [15]. It covers 52 of the 54 PDZ domains shared by both DomPep and MDSM and these 52 domains were therefore selected for the comparison of DomPep and MDSM. The performance of a model was evaluated by calculating the area under a receiver operating characteristic curve (AROC). The average AROC (0.81) of the 52 DomPep models was significantly greater than that (0.75) of the MDSM models (p-value = 7.9×10^−4^, Wilcoxon signed-rank test; Table S1). Additionally, we investigated whether the favorable performance of DomPep is due to the inclusion of more peptides from specificity-similar domains that were identified based on the DomPep strategy. To this end, we constructed models for individual PDZ domains using the experimentally-verified binding peptides (Table S1) and compared them with the related DomPep models. The above training and test sets were employed for this comparison. Indeed, the DomPep models had better accuracy (p-value = 7×10^−4^). Therefore, we concluded that the DomPep strategy leads to improved prediction accuracy.

Ideally a prediction method for domain-ligand interactions should be capable of identifying binding partners not only for domains included in the training set but also for family members not included in training. We therefore compared the performance of DomPep with that of SPSSM, a method that can be used for predicting binding partners of an untrained PDZ domain [25]. In this case, the independent test set was composed of interactions between human or worm PDZ domains and peptides identified from screening phage-displayed peptide libraries [13]. A query PDZ domain was aligned to the mouse PDZ domains in the training set in order to identify a matching mouse PDZ domain that share significant sequence identity with the query. Ten PDZ domains (four from human and six from worm) were found to have matching mouse PDZ domains with moderate DSI values (50–80%), and this group of PDZ domains was used as the final test set. For a query PDZ domain in the test set, we used peptides identified as specific binders from phage display as the positives and peptides that bound to other PDZ domains in the set, but not to query domain, as negatives. The average AROC of the DomPep models was greater than that of the SPSSM models (p-value = 4.5×10^−3^, Table S2). Because the same dataset was used to train both DomPep and SPSSM [25], our data suggests that DomPep is more accurate than SPSSM in identifying binding peptides for a PDZ domain that shares moderate sequence identity (e.g., DSI = 50–80%) to a domain included in the training set.

### Application of DomPep to the SH2 domain family

**Figure 3:**
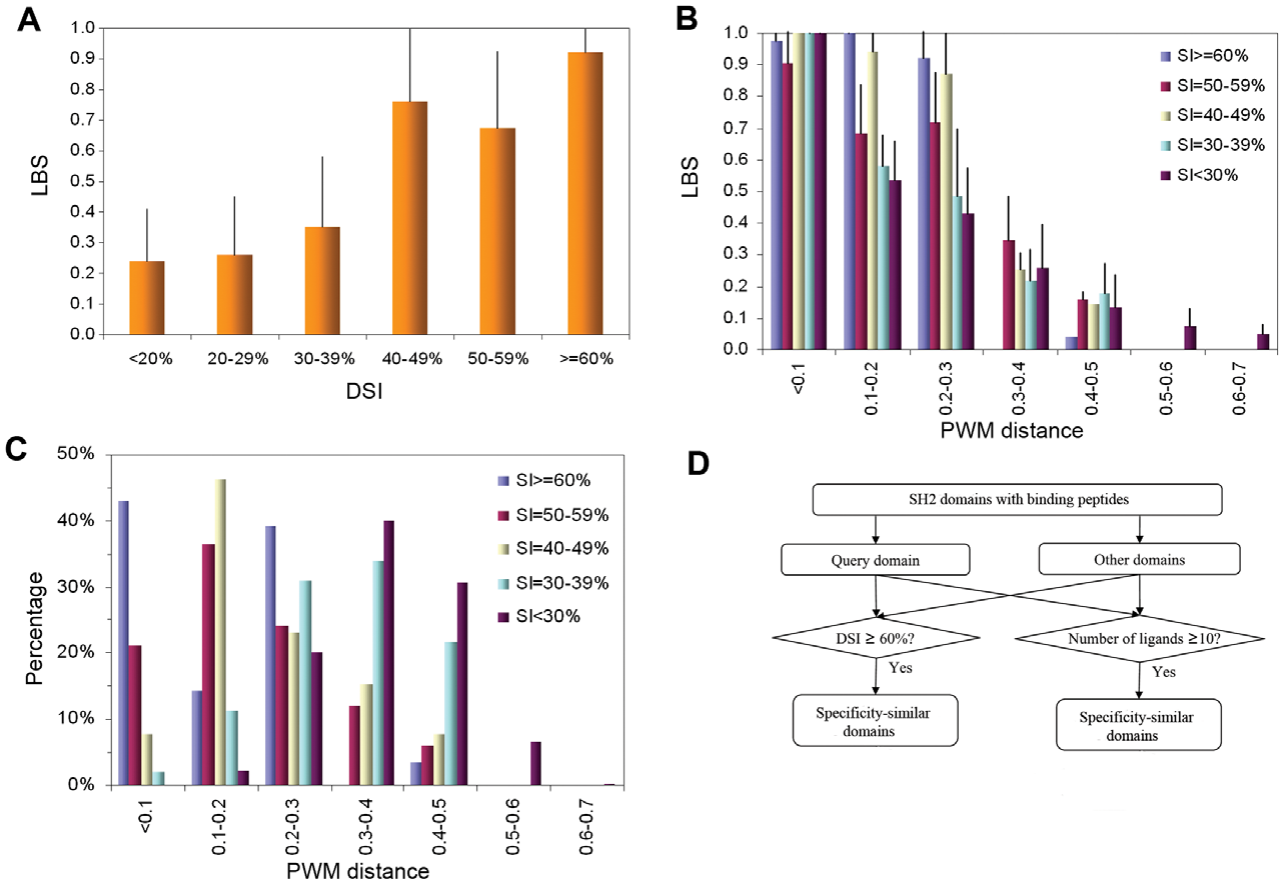
Correlation of LBS, DSI and PWM distance with each other and with the specificity of the SH2 domain. (A) Correlation of DSI and LBS in SH2 domain pairs based on domain-peptide array data. (B) Correlation of PWM distance and LBS for SH2 domain pairs within different DSI groups. It is apparent that LBS is related to both PWM distance and DSI. (C) The distribution of SH2 domain pairs along PWM distance for different DSI groups. (D) A strategy for the identification of similar domains for an SH2 domain.

The specificity of SH2 domains was systematically analyzed by probing an array of 6,200 phosphotyrosine-containing peptides respectively with 66 human SH2 domains [16]. This dataset was employed to build ANN models in NetPhorest, a collection of linear motifs recognized by phosphotyrosine-binding modules such as the SH2 and PTB domains [16]. The dataset of binding peptides was downloaded from http://netphorest.info/and used to evaluate the accuracy of DomPep in predicting SH2-peptide ligand interactions. We selected peptide sequences corresponding to residues from positions –2 to +4 with respect to the phosphotyrosine (pY) because these residues are sufficient in conferring specificity to most SH2 domains studies [12], [32], [33]. While in rare cases, such as in PLCg1, an SH2 domain may bind to a residue beyond P+4 [34] in addition to its recognition of the sequence within the -2 to +4 window, the vast majority of SH2 domains recognize residues in this window. We used 50 SH2 domains to screen oriented peptide array libraries with the degenerated sequence of XX-pY-XXXX (-2-pY-+4) or XXX-pY-XXXXXX (-4-pY-+6), where X represents a mixture of natural amino acids, but found no significant difference in binding patterns for an SH2 domain (Huang et al MCP, 2008; and unpublished data). Moreover, we extended the sequence coverage beyond the -2-pY-+4 motif in our model building, but found no significant improvement in predictive performance (data not shown). Since each of the 66 SH2 domains covered by the peptide-array experiment bound to more than 10 peptides [16], they were included in characterizing the relationships of the three parameters- LBS, DSI and PWM distance. As shown in Figure 3A, we calculated LBS and DSI for each SH2 domain pair and plotted them against each other. Domain pairs with DSI≥60% were found to have the highest average LBS (0.92). Significant difference was observed between pairs with DSI<40% and those with DSI>40% (p value = 5E-15, permutation test). The average LBS value (0.74) for the pairs with DSI = 40-49% is similar to those in the DSI = 50–59% group (LBS = 0.67), suggesting that for two SH2 domains that share 40-59% sequence identity, their binding peptides have an approximately 70% chance to overlap. Moreover, SH2 domains with low DSI values (<30%) share on average 25% of binding peptides. The standard deviation, however, is high (∼20%), indicating that the peptide-overlapping is spread out over a large range of values. Therefore, when a prediction model for a SH2 domain is constructed, the negative training peptides may not be selected from the pool of peptides bound to other SH2 domains. We therefore randomly chose peptides from the Swiss-Prot database as negatives to construct the SH2 models (see Methods for details).

We further investigated the relationship between PWM distance and LBS for SH2 domain pairs grouped according to DSI (Fig. 3B). A reverse correlation of LBS with PWM distance was noted, which is similar to that observed for the PDZ domains. However, when PWM distance is within the range of 0.1–0.3, a larger DSI value correlates with a greater LBS, and vice versa. It is also observed that all pairs in the DSI≥60% group had a PWM distance smaller than 0.3 and LBS greater than 0.9, except for the pair of SH2D3C and BCAR3 (DSI = 68%; PWM distance = 0.47). For the DSI = 40–59% group, a LBS value greater than 0.7 was obtained for pairs with PWM distance smaller than 0.3. We inspected the distribution of DSI groups along the PWM distance axis (Fig. 3C). In general, we found that domain pairs that share a high sequence identity possess smaller PWM distance. Specifically, 96% of SH2 domain pairs with DSI≥60% had PWM distance smaller than 0.3. This is in contrast to 80% of domain pairs in the DSI = 40–59% and 45% in the DSI<40% categories that had PWM distances smaller than 0.3. In addition, the PWM distance for SH2 domain pairs ranges from 0 to 0.7, which is narrower than for the PDZ domains (PWM = 0–0.9, Fig.1C), suggesting that SH2 domains may share binding peptides even when they are significantly different in sequences. Indeed, three SOCS2, SOCS3 and SOCS7 SH2 domains have DSI values of 20–30% but recognized almost the same peptides in the array [16].

Similar to PDZ domains, we considered SH2 pairs with LBS≥0.7 as having similar specificity. This threshold was used to determine the cutoffs for DSI and PWM distance, respectively. According to the above analysis (Fig. 3A–C), LBS≥0.7 corresponds to DSI≥60%, or PWM distance<0.1, or DSI≥40% and PWM distance<0.3 (Fig. 3D). We constructed models for SH2 domains using the same method as employed for the PDZ domains.

### Comparison of DomPep with ANN models in NetPhorest

We evaluated the performance of SH2 DomPep models by comparing them to Netphorest [16]. NetPhorest combines a domain-family phylogenetic tree with a backtracking algorithm to organize available in vitro and in vivo binding data and derive prediction models for domains. It currently contains prediction models for 179 kinases and 104 phosphorylation-dependent binding domains, including 93 SH2 domains [16]. NetPhorest comprises both ANN and PSSM models that are based on specificity data from pTyr-peptide array binding and OPAL screens [12], respectively. Since ANN models were constructed using the peptide array binding data that could be employed for the construction of DomPep models, we compared DomPep with the ANN models in NetPhorest.

The training set was based on the published SH2 domain-peptide array data [16]. The test set was based on binding data for eight randomly chosen human SH2 domains for which peptide arrays were employed to experimentally determine their specificities (Table S3). The average AROC of the DomPep models (0.71) was greater than that of the ANNs (0.61) (P value = 0.018, Wilcoxon signed-rank test). For the 8 SH2 domains tested, we found that DomPep models performed better than ANN models except for the GRB2 SH2 domain for which the same result was obtained from both methods (Table S3).

Several differences between the two methods may have contributed to the improved performance of DomPep over NetPhorest for the eight SH2 domains tested. For example, the two methods employ different strategies for the identification of specificity-similar domains. Furthermore, while DomPep randomly selected negative peptides of the training set from the Swiss-Prot protein sequence database, NetPhorest used positive peptides of other domains with small DSIs as negative training peptides for the query domain (Miller, et al., 2008). We found that the difference in performance originated largely from the different strategies by which the negative peptide training set was selected. DomPep showed comparable performance to NetPhorest when the same negative peptide sets were used as in the latter (data not shown). This is consistent with our observation (Figure 3A) that two SH2 domains with a small DSI value may share a non-negligible number of binding peptides.

### Application of DomPep models to predicting PDZ binding ligands on the genome scale

**Figure 4:**
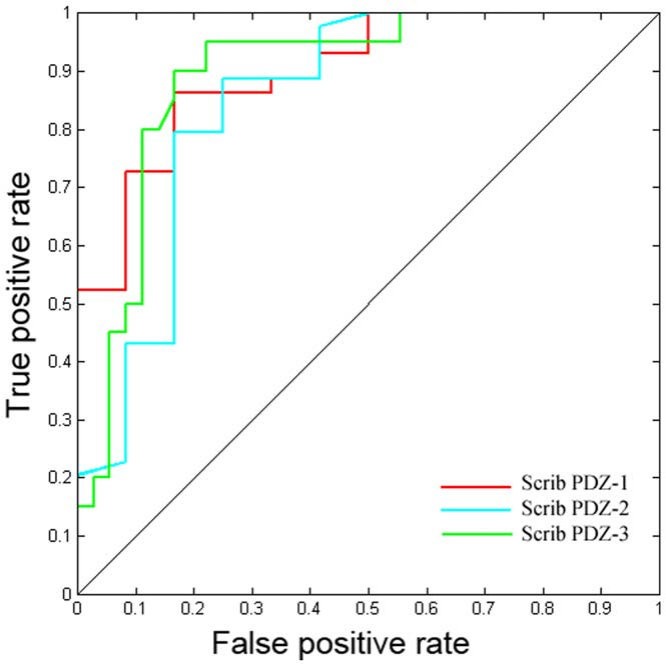
Application of DomPep to the prediction of PDZ-binding ligands. ROC curves of DomPep prediction for the three Scrib PDZ-1/2/3 domains using 56 peptides selected from the human protein database as the test set. The respective affinities of the three domains for the 56 peptides were measured by fluorescence polarization (Table 1).

Because DomPep compared favorably to MDSM in identifying ligands for a PDZ domain included in the training set and to SPSSM in identifying binding peptides for an untrained PDZ domain, we next tested it whether it could reliably PPIs mediated by a PDZ domain on the genome scale. To this end, we employed three DomPep models to predict interactions between the Scrib PDZ domains and human proteins from the Swiss-Prot protein database. The three prediction models, which were generated for the mouse Scrib PDZ-1, PDZ-2 and PDZ-3 domains, were trained with 6, 5 and 30 positive peptide, respectively (Table S1). Searching the protein database with these models yielded a list of potential PDZ ligands. We selected a group of 56 ligands with a wide range of prediction scores and measured their respective affinities (Kd) for the Scrib PDZ domains (Table 1). This experiment identified 14, 14, and 38 positives for the PDZ-1, -2, and -3 domains, respectively (Table 1). The AROC values for the three DomPep models were 0.90, 0.85 and 0.89, respectively, suggesting a high accuracy for identifying true positive interactions using our models (Figure 4). Importantly, we identified four peptides that were not included in the training set as physiological binders to the Scrib PDZ domains. The corresponding proteins, namely APC, LPP, VANGL2 and ZO2, were previously reported to interact with Scrib in vivo via its PDZ domains [35], [36], [37], [38], [39]. Our peptide-domain binding studies indicated that all four proteins are capable of binding to the PDZ-1 and PDZ-3 domains whereas two bind to PDZ-2 (Table 1). We standardized the prediction scores as Z scores based on the human protein database and found that the average Z score for the corresponding ten peptide-PDZ interactions (Z = 3.4) was greater than the average Z score for the whole set of positives (Z = 3.0) (Table 1). This result demonstrate the ability of the DomPep method in identifying physiological relevant interactions despite the fact that the prediction models were partly based on *in-vitro* array binding data that may contain non-specific binding events.

**Table 1:** Scrib PDZ1/2/3 domain-ligand interactions predicted by DomPep and the corresponding PDZ-peptide binding affinities.

Index	Protein name	Gene ID	C-termini	Kd (µM)			DomPep score (Z score)		
				PDZ1	PDZ2	PDZ3	PDZ1	PDZ2	PDZ3
1	ABR	NM_001092.3	RNTLYFSTDV	12.5 +/− 0.6	10.4 +/− 0.6	2.9 +/− 0.1	−0.09 (2.3)	0.3 (3.4)	−0.01 (2.3)
2	ACBD6	NM_032360.1	VLQRHTTGKA	NB	NB	NB	−1.39 (−1.2)	−1.36 (−1.2)	−2.6 (−1.7)
3	AHDC1	BC002677.1	PEDTFTVTSL	19.2 +/− 1.5	NB	10.4 +/− 0.6	0.43 (3.7)	0.32 (3.4)	0.31 (2.8)
4	ANKS4B	NM_145865.1	QPGQLVDTSL	NB	NB	28.2 +/− 2.0	0.58 (4.1)	0.43 (3.7)	0.31 (2.8)
5	APC*	NP_000029	HSGSYLVTSV	12.3 +/− 1.4	9.5 +/− 0.6	6.7 +/− 0.4	0.31 (3.4)	0.27 (3.3)	0.8 (3.6)
6	ARD50	BC024725.1	SFNYKKETPL	23.6 +/− 1.2	34.3 +/− 4.6	1.5 +/− 0.1	0.83 (4.7)	0.71 (4.5)	1.2 (4.2)
7	ARHGEF16	NM_014448.2	MERLRVETDV	27.5 +/− 2.4	7.7 +/− 0.3	2.1 +/− 0.1	0.75 (4.5)	1 (5.3)	1 (3.9)
8	BEGAIN	NM_020836.2	KAQLYGTLLN	NB	NB	NB	−1.91 (−2.6)	−1.94 (−2.8)	−2.2 (−1.1)
9	b-PIX	NP_003890	NDPAWDETNL	5.3 +/− 0.5	16.8 +/− 1.5	4.9 +/− 0.4	1 (5.2)	0.97 (5.2)	1 (3.9)
10	C11orf52	NM_080659.1	RYDSKNGTLV	NB	NB	26.5 +/− 2.4	−0.31 (1.7)	−0.07 (2.4)	0.16 (2.6)
11	C19orf57	BC012945.1	PRGDPPWREL	NB	NB	NB	−0.67 (0.7)	−0.69 (0.6)	−1.02 (0.7)
12	DIRAS1	NM_145173.1	DRVKGKCTLM	NB	NB	NB	−0.3 (1.7)	−0.24 (1.9)	−0.44 (1.7)
13	DSC54	NM_016644.1	ILRKSTTTTV	NB	NB	40.1 +/− 5.2	−0.21 (2)	0 (2.5)	−0.52 (1.5)
14	EPHA2	PV3688	DQVNTVGIPI	NB	NB	NB	−0.87 (0.2)	−0.84 (0.2)	−1.46 (0)
15	EPHA3	PV3359	TQSKNGPVPV	129 +/− 188	NB	NB	−1.04 (−0.3)	−0.89 (0.1)	−1.11 (0.6)
16	EPHA5	PV3840	VQLVNGMVPL	NB	75.7 +/− 35.0	45.6 +/− 8.7	−0.8 (0.4)	−0.84 (0.2)	−1.04 (0.7)
17	EPHA7	PV3689	LHLHGTGIQV	NB	NB	NB	−0.78 (0.4)	−0.58 (0.9)	−1.53 (−0.1)
18	EPHA8	PV3844	DPELEALHCL	NB	24.9 +/− 4.0	38.8 +/− 6.3	−0.73 (0.6)	−0.75 (0.5)	−1.19 (0.5)
19	FAM105B	NM_138348.3	PVRVCEETSL	1.9 +/− 0.14	17.6 +/− 2.0	3.4 +/− 0.2	0.88 (4.9)	0.66 (4.4)	1.24 (4.3)
20	FAM126B	NM_173822.1	SFNMQLISQV	NB	NB	39.3 +/− 5.1	−0.73 (0.6)	−0.73 (0.5)	−0.42 (1.7)
21	FLT1	NM_002019.1	SVVLYSTPPI	NB	NB	NB	−1.25 (−0.8)	−1.24 (−0.9)	−1.76 (−0.4)
22	FOXl1	NM_144769.1	VLYPREGTEV	NB	24.7 +/− 2.7	11.7 +/− 0.8	−0.19 (2)	0.07 (2.7)	0.31 (2.8)
23	GLO1	BC001741.1	LNPNKMATLM	NB	NB	59.5 +/− 6.6	−0.45 (1.3)	−0.38 (1.5)	−0.36 (1.8)
24	KCNA6	NM_002235.2	YAEKRMLTEV	NB	NB	34.8 +/− 3.6	−0.02 (2.5)	0.21 (3.1)	0.51 (3.1)
25	KCNJ10	BC034036.1	SALSVRISNV	NB	NB	13.2 +/− 1.0	−0.56 (1)	−0.37 (1.5)	−0.04 (2.3)
26	KIRREL2	BC007312.1	PSHPRLQTHV	NB	NB	21.3 +/− 2.4	0.33 (3.4)	0.36 (3.5)	0.08 (2.5)
27	LIMD1	NM_014240.1	SSTALHQHHF	NB	NB	NB	−0.68 (0.7)	−0.68 (0.7)	−1.92 (−0.7)
28	LPP*	NP_005569	VLTAKASTDL	24.2 +/− 3.9	NB	11.3 +/− 0.5	0.02 (2.6)	0.2 (3.1)	−0.13 (2.1)
29	MAPK12	PV3654	GARVSKETPL	20.3 +/− 2.0	56.1 +/− 10.1	1.7 +/− 0.1	0.83 (4.7)	0.71 (4.5)	1.2 (4.2)
30	MCM7	BC009398.1	NASRTRITFV	NB	NB	24.1 +/− 2.4	−0.07 (2.3)	0.13 (2.9)	0.41 (3)
31	MPG	BC014991.1	DRVAEQDTQA	NB	NB	186 +/− 51	−0.31 (1.7)	−0.22 (1.9)	−0.76 (1.1)
32	MTERFD1	NM_015942.3	QDFEKFLKTL	NB	NB	NB	−0.65 (0.8)	−0.71 (0.6)	−1.39 (0.2)
33	MUSK	PV3834	CERAEGTVSV	NB	NB	143 +/− 43.1	−0.75 (0.5)	−0.74 (0.5)	−1.01 (0.8)
34	PACAP	BC021275.1	EKVSATREEL	NB	NB	88.1 +/− 29.5	−1.01 (−0.2)	−0.84 (0.2)	−1.4 (0.1)
35	PDGFRA	NM_002609	PRAEAEDSFL	NB	NB	52.0 +/− 7.1	−0.84 (0.3)	−0.84 (0.2)	−0.32 (1.8)
36	PDGFRB	NM_002609	PRAEAEDSFL	NB	NB	59.6 +/− 11.9	−0.84 (0.3)	−0.84 (0.2)	−0.32 (1.8)
37	PRKCA	P2227	FVHPILQSAV	17.4 +/− 2.1	46.0 +/− 3.7	9.8 +/− 0.5	−0.51 (1.2)	−0.49 (1.2)	−0.14 (2.1)
38	PRKCB1	P2281	YTNPEFVINV	NB	NB	NB	−0.57 (1)	−0.34 (1.6)	−0.71 (1.2)
39	PSMA8	BC042820.1	AEKKKSKKSV	NB	NB	177 +/− 85.9	−0.43 (1.4)	−0.41 (1.4)	−1.35 (0.2)
40	PTE1	NM_005469.2	VKPQVSESKL	NB	NB	NB	−0.28 (1.8)	−0.44 (1.3)	0.28 (2.8)
41	RASL11B	NM_023940.1	SAKVRTVTSV	NB	NB	30.0 +/− 3.5	0.32 (3.4)	0.43 (3.7)	0.36 (2.9)
42	RPS6KA1	NM_001006665	RVRKLPSTTL	NB	NB	27.0 +/− 3.2	−0.06 (2.4)	−0.05 (2.4)	−0.39 (1.7)
43	RPS6KA2	NM_001006932	GMKRLTSTRL	NB	62.7 +/− 19.8	25.2 +/− 2.1	−0.02 (2.5)	−0.01 (2.5)	−0.31 (1.9)
44	SRC	NM_005417.3	EPQYQPGENL	NB	NB	48.6 +/− 4.4	−0.77 (0.5)	−0.74 (0.5)	−1.55 (−0.1)
45	STK16	BC053998.1	PAPGQHTTQI	NB	NB	52.2 +/− 5.6	−0.45 (1.3)	−0.38 (1.5)	−0.49 (1.6)
46	STK29	BC024291.1	KVATSYESSL	NB	NB	3.2 +/− 0.2	0.29 (3.3)	0.01 (2.6)	0.59 (3.3)
47	SYNJ2BP	BC007704.1	WAFMRYRQQL	NB	NB	32.0 +/− 1.8	−0.81 (0.4)	−0.68 (0.7)	−1.82 (−0.5)
48	TANK	NM_133484.1	VDIASAESSI	102 +/− 40	NB	13.6 +/− 0.6	−0.08 (2.3)	−0.29 (1.7)	0.49 (3.1)
49	TBK1	PV3504	DGGLRNVDCL	77 +/− 21.1	NB	67.8 +/− 16.5	−0.76 (0.5)	−0.75 (0.5)	−0.49 (1.6)
50	TPM2	NM_003289.3	DNALNDITSL	NB	NB	54.4 +/− 11.1	0.6 (4.1)	0.48 (3.9)	0.25 (2.7)
51	TRIM21	NM_003141.2	NIGSQGSTDY	NB	NB	116 +/− 24.0	−0.68 (0.7)	−0.43 (1.4)	−0.74 (1.2)
52	UBXD1	NM_025241.1	ELLSAIEKLL	NB	NB	NB	−0.03 (2.4)	−0.22 (1.9)	−0.38 (1.7)
53	VANGL2*	NP_065068	VMRLQSETSV	21.6 +/− 4.6	8.7 +/− 0.5	8.8 +/− 0.5	0.86 (4.8)	0.83 (4.8)	1.25 (4.3)
54	ZADH2	NM_175907.3	ELPHSVNSKL	NB	NB	NB	−0.63 (0.8)	−0.69 (0.6)	−0.99 (0.8)
55	ZNF654	NM_018293.1	SSAQPSETIL	NB	32.1 +/− 4.6	18.3 +/− 1.0	0.6 (4.1)	0.48 (3.9)	0.91 (3.8)
56	ZO2*	NP_004808	QSARYDTEL	16.0 +/− 2.5	NB	13.7 +/− 0.6	0.06 (2.7)	0.08 (2.8)	0.2 (2.6)

a K_d_ value of 100 µM was applied as the binding cutoff, referred from previous study ^15^.

*Proteins that have been identified to interact with Scrib protein in vivo via its PDZ domains.

### Framework of the DomPep server

**Figure 5:**
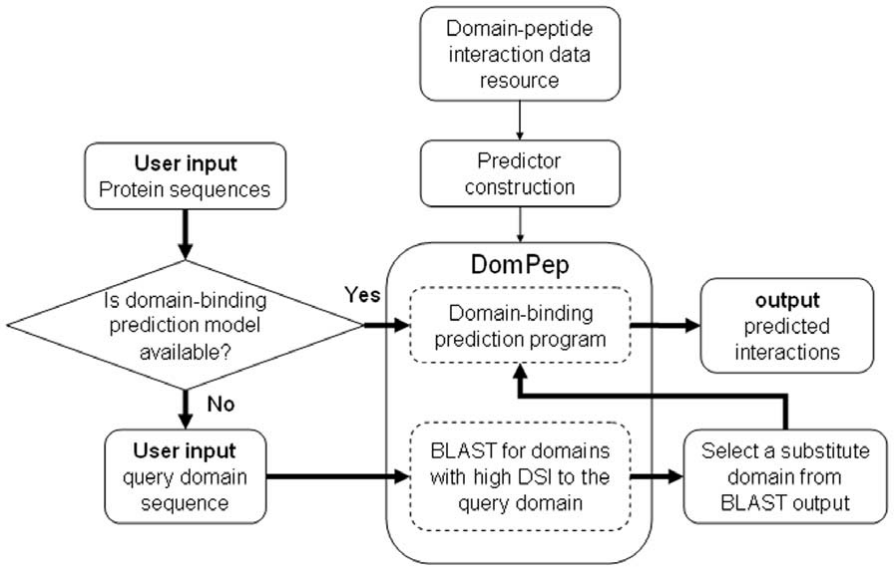
Framework of the DomPep program. DomPep contains three components- a Graphical user interface (GUI)**,** a local BLAST server and a set of embedded domain-peptide binding predictors. The GUI allows a user to input protein sequences and choose predictors for specific domains. If the query domain does not have an embedded predictor, a substitute query domain with a known predictor can be identified from domains that exhibit the highest DSI from a BLAST search. The output of a DomPep prediction consists of a list of peptides with prediction scores arranged in a descending order from top to bottom. The corresponding proteins are listed in a separate column.

To ensure that the DomPep method is freely accessible to the signaling community, we created the first online version of the DomPep server (http://lilab.uwo.ca/DomPep.html) to predict binding ligands for the PDZ and SH2 domains (Figure 5). The server comprises two core components: model construction and user interface. The online models were built using all available interaction data for the PDZ or SH2 domains from current PPI databases (e.g., PDZbase for the PDZ domain and phospho.ELM for the SH2 domain) (Figure 1E) [12], [13], [15], [16], [19], [30], [40]. The pool of positive peptides used in training a model is expanded according to the strategy illustrated in Fig. 2D and Fig. 3D. The current version of DomPep contains 174 PDZ models and 87 SH2 models (covering 97 SH2 domains). The BLAST search program linked to the server allow onsite sequence alignment to identify domains that are similar to the query domain when necessary [24]. The user may input a query protein sequence and select for the domain of interest (i.e. PDZ or SH2). If the query domain has a built-in DomPep model, potential interactions will be predicted and listed in the output file. If no built-in model exists for the query domain, the sequence of the query domain will be used as input in BLAST to find homologous or similar domains (ranked according to DSI values) that have DomPep models. The top 10 domains with DSI≥40% are displayed for the user to select as substitutes for the query domain. Our study suggests that a substitute model can be used for PDZ domains with DSI≥50% and for SH2 domains with DSI≥60%. DomPep provides ‘high’, ‘medium’ and ‘low’ stringency prediction, corresponding to true positive rate of 70%, 50% and 30%, respectively, in the average ROC curve generated from data obtained for PDZ or SH2 domain family (Tables S1 and S3).

## Discussion

We described a novel method called DomPep based on sequence information and experimental data to predict PPIs that involve the recognition of linear peptide motifs by modular domains. Unlike existing sequence-based methods in which a common model is constructed for a group of domains, the DomPep method contains models for individual domains constructed from experimental data of the query domain and/or specificity-similar domains identified by sequence identity and/or closeness in specificity profiles. DomPep employs a novel classification strategy to identify domains that are “similar”. Two parameters, namely DSI and PWM distance, are used together to identify specificity-similar domains based on experimentally verified binding peptides. The threshold values of DSI and PWM distances are calibrated against LBS, or ligand binding specificity, obtained from domain-peptide array binding data. To ensure the accuracy of calibration, a LBS value is derived only from domain pairs each of which has more than 10 positive peptides. To maximize the dataset for prediction model training, peptides that bind specifically to either the query domain or specificity-similar domains that display a greater-than-the-threshold value of DSI or PWM distance are pooled to form the positive peptide set for the query domain.

While DomPep compares favorably in identifying PDZ- or SH2-binding ligands to existing algorithms, it should be cautioned that a few limitations may affect the performance of DomPep models. First, DomPep depends on large-scale domain-peptide array interaction data. However, this problem will become less of a concern as peptide and protein arrays are becoming increasingly popular and the reservoir of proteomic data is rapidly increasing in size with the application of these and other proteomic technologies to the study of PPIs. Second, unlike NetPhorest that makes use of all forms of array-binding data [41], DomPep is limited to using data from peptide array-domain binding experiments only. Although this problem is difficult to solve given the specific parameters (ie., LBS, PWM distances) that have to be defined, future versions of DomPep should seek ways by which to explore all forms of experimental data for model training and testing. It should be noted also that the current version of DomPep cannot be used to predict binding partners for a mutated domain with significantly altered specificity. In this regard, structure-based methods would be more suitable for ligand identification if the mutated residues are located at the binding interface. We expect that DomPep, when used in conjunction with other sequence- or structure-based algorithms, would provide a powerful tool for *in silico* identification of domain-ligand interactions.

To the best of our knowledge, DomPep contains the largest number of prediction models for modular interaction domains in all the algorithms published to date. Although the current version of DomPep is limited to predicting PPIs mediated by the PDZ or SH2 domains, the general strategy we described here may be applied to other protein-interaction domains such as SH3, WW, and FHA [4], [5], [8]. There are approximately 261 PDZ domains and 120 SH2 domains in the human genome. Together with other interaction domains, they represent a prevalent feature of the human proteome. Because of their pivotal roles in regulating cellular functions [8], understanding how these domains are used to connect proteins in a specific signaling pathway or to form elaborate PPI networks is a key to understanding normal cell physiology and the molecular basis of human diseases [42], [43]. We expect DomPep to provide a useful tool to suggest novel PPIs for experimental validation and for large-scale network analysis.

## Materials and Methods

### Ligand Binding Similarity (LBS)

We created a parameter, LBS, to measure relative specificity between two domains based on interaction data from the same peptide pool (i.e. domain- or peptide-array data). LBS is defined as the percentage of peptides commonly selected by a domain pair *i* and *j* as in, 
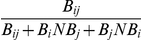



Where B*_ij_* is the number of peptides selected by both domains *i* and *j*, B*_i_*NB*_j_* is the number of peptides that bind to domain *i* but not to domain *j*. LBS ranges from 0 to 1, where “1” indicates that both domains have the same set of binding and non-binding peptides, or identical specificity. This formula, suitable for processing the MacBeath dataset on the PDZ domain, needs to be modified to accommodate the SH2 domain-peptide array binding data used in NetPhorest [16]. In the SH2 domain-peptide array binding data, the ABL1 SH2 domain bound to four times more peptides than the ABL2 SH2 domains (i.e. 173 binders for the former compared to 40 for the latter), due likely to variations in signal strength between the two binding experiments. If we counted the common ligands and calculated the LBS according to the above formula, a low LBS value of 0.23 would have been produced. However, this is unlikely to be true. Since the two SH2 domains share 91% sequence identity and that every ligand of the ABL2 SH2 domain was also recognized by the ABL1 SH2 domain, they must have identical specificity. This assertion is also supported by data from oriented peptide array library screening [12]. In the NetPhorest program the two domains were considered as similar and the binding peptides for both domains were combined to build a single model [16]. To resolve this contradiction, we modify the LBS formula for the SH2 domain as 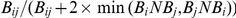



According to this formula, the LBS value for the ABL1 and ABL2 SH2 domains increases to 1 ( =  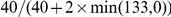), indicating identical specificity.

### Position weight matrix (PWM) distance

PWM distance was used to measure the difference in specificity between two domains. The PWM was calculated by aligning the amino acid sequence of all binding peptides for a domain, where the element PWM*_i,j_* contains the percentage of amino acid residue *j* at position *i* in the aligned peptides. The PWM distance between domains A and B is calculated using the formula 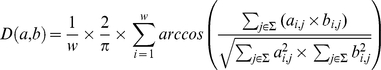
where a = PWM for domain A. b = PWM for domain B. Σ = 20 amino acids used in PWM. w = number of columns in the PWM. For instance, w is 5 (corresponding to residues P-4 to P0) for PDZs and 6 (corresponding to pY-2 to pY+4 except pY) for SH2s.

The PWM distance ranges from 0 to 1, where 0 represents identical PWMs and suggests identical specificity. In order to derive accurate PWM distance, domains with fewer than 10 binding peptides were excluded from the analysis.

In this formula, we took inverse cosine of the inner product of the two unit vectors which is equivalent to the angle between the two unit vectors. We did not use the inner product directly because it is the cosine of the angle which will excessively reward very similar vectors and excessively penalize dissimilar vectors.

### Construction of DomPep models

We used the Support Vector Machine (SVM) program SVMlight with the conventional linear kernel to build prediction models [44]. According to previous analyses [12], [15], [32], the DomPep model for a PDZ domain took into account of five C-terminal residues of the peptide ligands and the model for an SH2 domain was derived for six residues (from pY-2 to pY+4, pY set as residue “0”) of the peptide. Residues at each position were coded using the conventional orthogonal method by 20 dimensions of 0-1 vectors [28]. Consequently, peptides for PDZ domains were coded by 100-dimension binary vectors while peptides for SH2s were coded by 120-dimension vectors. We constructed models for domains with at least three positive ligands in the training set.

### Peptide arrays for SH2 domain binding analysis

Peptide arrays were synthesized following established protocols [19]. A total of 720 pY-containing peptides covering positions -2 to +4 with respect to the pY were arrayed on cellulose membranes. The printed membranes were incubated, respectively, with four SH2 domains (i.e., HSH2D, TNS4, GRB7 and GADS) fused to glutathione S-transferase (GST) or with the GST protein, used as a negative control. After washing, the bound domains were detected by an anti-GST Far-Western blot. After background subtraction and removal of 18 GST-binding peptides identified as false positives, each binding experiment yielded 702 data points that form the peptide recognition profile of a given SH2 domain (Figure S2, Table S4). The same strategy used previously to differentiate between binding and nonbinding peptides [19] was applied to the four domains. A spot value cutoff of 80/50/120 was used for the GADS/GRB7/HSH2D SH2 domains, respectively.

## Supporting Information

Figure S1 Phylogenetic tree of the human PDZ Domains. 5 pairs of domains with similar specificities selected as example are shown with different colors. They are the first PDZ domain in protein ZO2 (ZO2/1) and HtrA3 with SI = 15% (pink), DLG3/2 and MAGI3/2 with SI = 17% (grey), SHANK1 and PDZK1/1 with SI = 23% (blue), MAGI1/5 and MAGI3/4 with SI = 24% (green), LRRC7 and SCRIB/2 with SI = 36% (red). The first three pairs are identified by DomPep which have BS>0.7 based on PDZ domain arrays [15] and the rest are identified elsewhere [13]. It is noted that MAGI proteins are detected by SMART to contain 6 PDZ domains which are shown in this figure [45]. The figure is prepared with iTOL [46].
(TIF)

Figure S2 Respective binding profiles for the SH2 domains from HSH2D (A), GRB7 (B), GADS (C) and for control GST (D) on arrays of phosphotyrosine-containing peptide ligands. These peptides are derived from the PhosphoSite database and were predicted to be highly connected to the four SH2 domains using SMALI program [19]. The sequences of the peptides and the binding signal values are provided in Tables S4.
(TIF)

Table S1 Comparison of DomPep with MSMD on predicting PDZ domain-ligand interactions.
(DOC)

Table S2 Comparison of DomPep with SPSSM for ability to predict PDZ domain-ligand interactions for PDZ domains in other species than *Mus musculus*.
(DOC)

Table S3 Comparison of DomPep with Netphorest with ANN models on predicting SH2 domain-ligand interactions.
(DOC)

Table S4 Peptides tested for binding the SH2 domains of GADS, GRB7 and HSH2D in the peptide array experiment.
(DOC)
