# Supplementary figures and images for: DomPep—A General Method for Predicting Modular Domain-Mediated Protein-Protein Interactions
Source: PLoS One. 2011 Oct 7;6(10):e25528. doi: 10.1371/journal.pone.0025528 (PMC3189207; doi:10.1371/journal.pone.0025528)

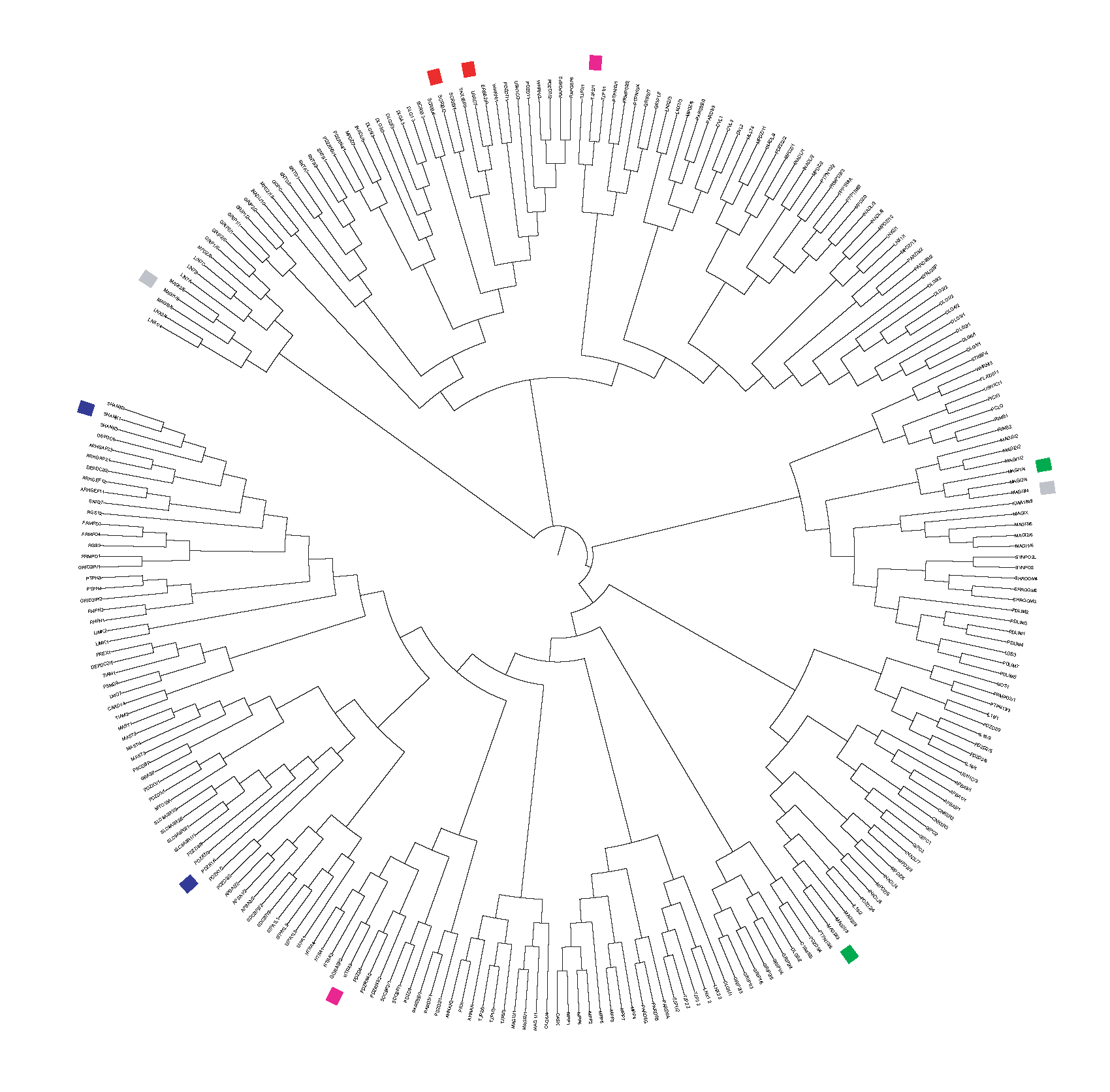

Supplement: Figure S1 — Phylogenetic tree of the human PDZ Domains. 5 pairs of domains with similar specificities selected as example are shown with different colors. They are the first PDZ domain in protein ZO2 (ZO2/1) and HtrA3 with SI = 15% (pink), DLG3/2 and MAGI3/2 with SI = 17% (grey), SHANK1 and PDZK1/1 with SI = 23% (blue), MAGI1/5 and MAGI3/4 with SI = 24% (green), LRRC7 and SCRIB/2 with SI = 36% (red). The first three pairs are identified by DomPep which have BS>0.7 based on PDZ domain arrays [15] and the rest are identified elsewhere [13]. It is noted that MAGI proteins are detected by SMART to contain 6 PDZ domains which are shown in this figure [45]. The figure is prepared with iTOL [46]. (TIF) [file pone.0025528.s001.tif]

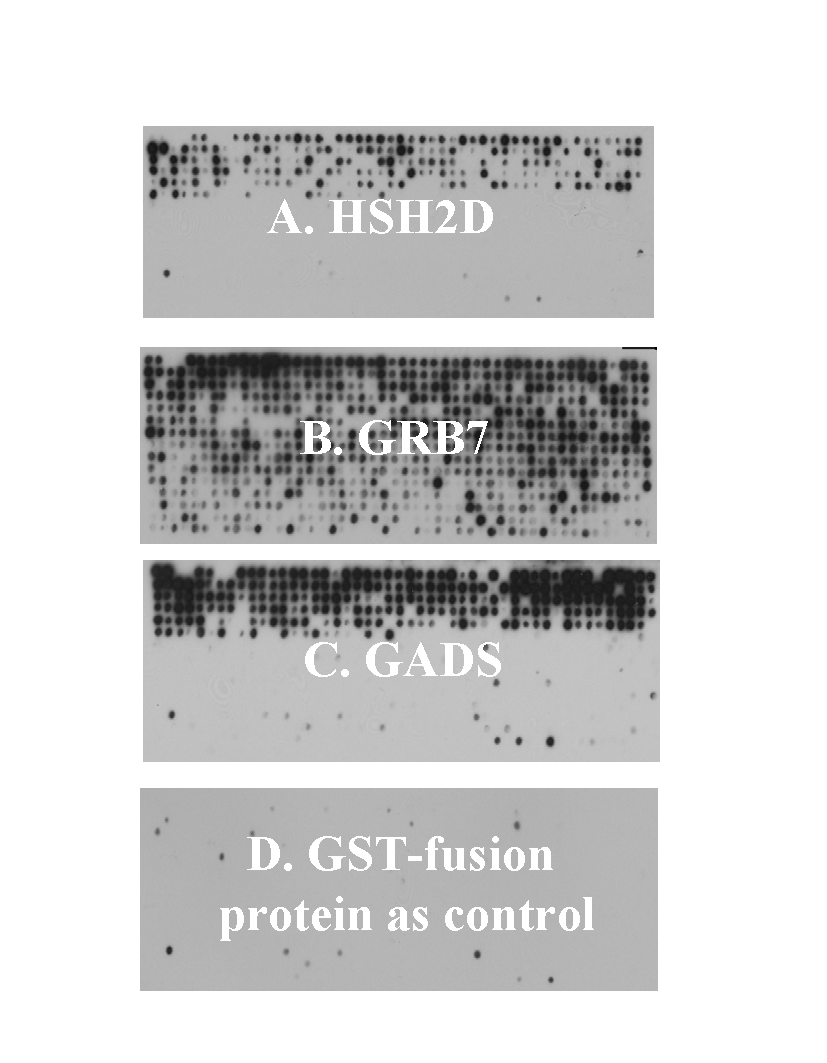

Supplement: Figure S2 — Respective binding profiles for the SH2 domains from HSH2D (A), GRB7 (B), GADS (C) and for control GST (D) on arrays of phosphotyrosine-containing peptide ligands. These peptides are derived from the PhosphoSite database and were predicted to be highly connected to the four SH2 domains using SMALI program [19]. The sequences of the peptides and the binding signal values are provided in Tables S4. (TIF) [file pone.0025528.s002.tif]
