# Supplementary material for: DomPep—A General Method for Predicting Modular Domain-Mediated Protein-Protein Interactions
Source: PLoS One. 2011 Oct 7;6(10):e25528. doi: 10.1371/journal.pone.0025528 (PMC3189207; doi:10.1371/journal.pone.0025528)
Supplement: Table S1 — Comparison of DomPep with MSMD on predicting PDZ domain-ligand interactions. (DOC) [file pone.0025528.s003.doc]

**Table S1.** Comparison of DomPep with MSMD on predicting PDZ domain-ligand interactions*

| **Index** | **Domain name** | **Positives in the training set** | **Positives (DomPep) in the traing set** | **AROC**  **DomPep** | **AROC MSMD** |
| --- | --- | --- | --- | --- | --- |
| 1 | α1-syntrophin (1/1) | 6 | 16 | 0.94 | 0.91 |
| 2 | β1-syntrophin (1/1) | 15 | 41 | 0.95 | 0.92 |
| 3 | Cipp (10/10) | 3 | 3 | 0.47 | 0.71 |
| 4 | Cipp (5/10) | 5 | 5 | 0.87 | 0.97 |
| 5 | Cipp (8/10) | 10 | 10 | 1.00 | 1.00 |
| 6 | Cipp (9/10) | 8 | 8 | 0.74 | 0.27 |
| 7 | Dvl1 (1/1) | 3 | 4 | 0.51 | 0.59 |
| 8 | Dvl2 (1/1) | 2 | 4 | 0.56 | 0.63 |
| 9 | Dvl3 (1/1) | 4 | 4 | 0.46 | 0.73 |
| 10 | Erbin (1/1) | 2 | 4 | 0.87 | 0.81 |
| 11 | γ2-syntrophin (1/1) | 4 | 19 | 1.00 | 0.82 |
| 12 | Gm1582 (2/3) | 16 | 47 | 0.87 | 0.78 |
| 13 | GRASP55 (1/1) | 5 | 5 | 0.75 | 0.64 |
| 14 | Grip2 (5/7) | 6 | 6 | 0.94 | 0.68 |
| 15 | HtrA1 (1/1) | 41 | 41 | 0.59 | 0.62 |
| 16 | HtrA3 (1/1) | 29 | 30 | 0.72 | 0.69 |
| 17 | Interleukin 16 (1/4) | 5 | 5 | 0.88 | 0.83 |
| 18 | LARG (1/1) | 3 | 3 | 0.81 | 0.77 |
| 19 | LIN-7A (1/1) | 3 | 19 | 0.90 | 0.82 |
| 20 | Lin7c (1/1) | 19 | 44 | 0.93 | 0.88 |
| 21 | Lrrc7 (1/1) | 3 | 4 | 0.19 | 0.13 |
| 22 | Magi-1 (2/6) | 2 | 17 | 0.86 | 0.31 |
| 23 | Magi-1 (6/6) | 12 | 33 | 0.84 | 0.76 |
| 24 | Magi-2 (5/6) | 2 | 2 | 0.95 | 0.93 |
| 25 | Magi-2 (6/6) | 13 | 31 | 0.98 | 0.68 |
| 26 | Magi-3 (5/5) | 25 | 44 | 0.90 | 0.79 |
| 27 | MUPP1 (10/13) | 8 | 14 | 1.00 | 1.00 |
| 28 | MUPP1 (13/13) | 5 | 14 | 0.72 | 0.89 |
| 29 | MUPP1 (5/13) | 4 | 5 | 0.97 | 0.79 |
| 30 | NHERF-1 (1/2) | 3 | 6 | 0.93 | 0.92 |
| 31 | NHERF-2 (2/2) | 4 | 6 | 0.95 | 0.51 |
| 32 | PAR3B (1/3) | 3 | 3 | 0.45 | 0.64 |
| 33 | PDZ-RGS3 (1/1) | 14 | 13 | 0.76 | 0.79 |
| 34 | Pdzk1 (1/4) | 11 | 21 | 0.76 | 0.64 |
| 35 | Pdzk1 (3/4) | 7 | 6 | 0.92 | 0.91 |
| 36 | Pdzk3 (1/1) | 20 | 19 | 0.38 | 0.34 |
| 37 | PSD95 (1/3) | 11 | 27 | 0.82 | 0.81 |
| 38 | PTP-BL (2/5) | 20 | 37 | 0.94 | 0.95 |
| 39 | SAP102 (3/3) | 9 | 13 | 0.97 | 0.84 |
| 40 | SAP97 (1/3) | 13 | 29 | 0.88 | 0.80 |
| 41 | SAP97 (3/3) | 13 | 36 | 0.92 | 0.88 |
| 42 | Scrb1 (1/4) | 5 | 6 | 0.91 | 0.89 |
| 43 | Scrb1 (2/4) | 3 | 5 | 0.92 | 0.86 |
| 44 | Scrb1 (3/4) | 21 | 30 | 0.90 | 0.86 |
| 45 | Semcap3 (1/2) | 7 | 7 | 0.97 | 0.97 |
| 46 | Shank1 (1/1) | 17 | 21 | 0.93 | 0.85 |
| 47 | Shank3 (1/1) | 18 | 21 | 0.93 | 0.78 |
| 48 | Shroom (1/1) | 3 | 3 | 0.79 | 0.85 |
| 49 | TIP-1 (1/1) | 6 | 5 | 0.84 | 0.79 |
| 50 | ZO-1 (1/3) | 20 | 30 | 0.81 | 0.61 |
| 51 | ZO-2 (1/3) | 13 | 30 | 0.73 | 0.40 |
| 52 | ZO-3 (1/3) | 1 | 14 | 0.72 | 0.72 |
| **Average of AROCs** | |  |  | **0.81** | **0.75** |
| ***p*-value** | |  |  | **7.9x10^-4^** | |

**Footnote*. The two methods were benchmarked using the same independent test set previously used to test MSMD . Together 52 predictors were compared. The second column shows protein name followed by the specific PDZ domain number and the total number of PDZ domains in the protein. For instance, ZO-1 (1/3) denotes the first PDZ domain in the protein ZO-1 that contains a total of 3 PDZ domains. The third column lists the number of positive binding peptides in the training set of MSMD . The number of positives used for training in DomPep, expanded in most cases according to the method described in the main text, is listed in the fourth column. The area under the receiver operating characteristic curve (AROC) is calculated to evaluate the performance of the predictor. The averages of AROCs for each method are listed at the bottom together with the *p*-values calculated by paired paired Wilcoxon signed rank test.

References:

1. Stiffler MA, Chen JR, Grantcharova VP, Lei Y, Fuchs D, et al. (2007) PDZ domain binding selectivity is optimized across the mouse proteome. Science 317: 364-369.
