# Supplementary material for: DomPep—A General Method for Predicting Modular Domain-Mediated Protein-Protein Interactions
Source: PLoS One. 2011 Oct 7;6(10):e25528. doi: 10.1371/journal.pone.0025528 (PMC3189207; doi:10.1371/journal.pone.0025528)
Supplement: Table S2 — Comparison of DomPep with SPSSM for ability to predict PDZ domain-ligand interactions for PDZ domains in other species than Mus musculus. (DOC) [file pone.0025528.s004.doc]

**Table S2**. Comparison of DomPep and SPSSM for ability to predict PDZ domain-ligand interactions for PDZ domains in other species than *Mus musculus**

| **index** | **Domain name** | **Species** | **AROC**  **SPSSM** | **Domain substitute used in DomPep** | **sequence identity** | **AROC**  **DomPep** |
| --- | --- | --- | --- | --- | --- | --- |
| 1 | MAGI1-4 | *Homo Sapiens* | 0.22 | Magi-2(5/6) | 71% | 0.45 |
| 2 | C34F11.9a-1 | *C. elegans* | 0.64 | Dvl3(1/1) | 71% | 0.69 |
| 3 | MPDZ-12 | *Homo Sapiens* | 0.93 | Cipp(10/10) | 66% | 0.95 |
| 4 | C52A11.4-8 | *C. elegans* | 0.64 | Cipp(8/10) | 63% | 0.95 |
| 5 | C25F6.2a-3 | *C. elegans* | 0.90 | SAP102(3/3) | 60% | 0.95 |
| 6 | DLG1-2 | *Homo Sapiens* | 0.87 | PSD95(1/3) | 55% | 0.88 |
| 7 | C52A11.4-9 | *C. elegans* | 0.57 | Cipp(9/10) | 55% | 0.87 |
| 8 | DLG3-2 | *Homo Sapiens* | 0.84 | SAP97(1/3) | 53% | 0.88 |
| 9 | K01A6.2-5 | *C. elegans* | 0.93 | Magi-3(5/5) | 51% | 0.95 |
| 10 | C33B4.3-1 | *C. elegans* | 0.83 | Shank1(1/1) | 50% | 0.83 |
| **Average of domains with SI (50-80%) to PDZ domains included in DomPep** | | | **0.74** |  |  | **0.84** |
| ***p*-value** | |  | **4.5x10^-3^** | | | |

**Footnote*. The test set contains 6 PDZ domains from *C. elegans* and 4 PDZ domains from *Homo Sapiens* whose binding peptides were identified using phage display experiment . The fifth column lists domains that have DomPep predictors and have the highest sequence identity to the domains in the test set. The sequence identity is shown in the sixth column.

References:

1. Chen JR, Chang BH, Allen JE, Stiffler MA, MacBeath G (2008) Predicting PDZ domain-peptide interactions from primary sequences. Nat Biotechnol 26: 1041-1045.

2. Tonikian R, Zhang Y, Sazinsky SL, Currell B, Yeh JH, et al. (2008) A specificity map for the PDZ domain family. PLoS Biol 6: e239.
