# Supplementary material for: DomPep—A General Method for Predicting Modular Domain-Mediated Protein-Protein Interactions
Source: PLoS One. 2011 Oct 7;6(10):e25528. doi: 10.1371/journal.pone.0025528 (PMC3189207; doi:10.1371/journal.pone.0025528)
Supplement: Table S3 — Comparison of DomPep with Netphorest with ANN models on predicting SH2 domain-ligand interactions. (DOC) [file pone.0025528.s005.doc]

**Table S3.** Comparison of DomPep to Netphorest with ANN models on predicting SH2 domain-ligand interactions*

| **Index** | **Protein name** | **AROC**  **Netphorest (ANN model)** | **AROC**  **DomPep** |
| --- | --- | --- | --- |
| 1 | BRDG1 | 0.64 | 0.68 |
| 2 | CRK | 0.35 | 0.40 |
| 3 | FGR | 0.58 | 0.60 |
| 4 | GRB2 | 0.93 | 0.93 |
| 5 | GADS | 0.64 | 0.83 |
| 6 | GRB7 | 0.46 | 0.67 |
| 7 | HSH2D | 0.71 | 0.85 |
| **Average of domains predicted by ANN models** | | **0.61** | **0.71** |
| ***p*-value** |  | **0.018** | |

*Footnote. The independent test set contains nine SH2 domains where the data are derived from literature or listed in Table S4.

References:

1. Miller ML, Jensen LJ, Diella F, Jørgensen C, Tinti M, et al. (2008) Linear motif atlas for phosphorylation-dependent signaling. Sci Signal 1: ra2.

2. Li L, Wu C, Huang H, Zhang K, Gan J, et al. (2008) Prediction of phosphotyrosine signaling networks using a scoring matrix-assisted ligand identification approach. Nucleic Acids Res 36: 3263-3273.
