# Supplementary material for: DomPep—A General Method for Predicting Modular Domain-Mediated Protein-Protein Interactions
Source: PLoS One. 2011 Oct 7;6(10):e25528. doi: 10.1371/journal.pone.0025528 (PMC3189207; doi:10.1371/journal.pone.0025528)
Supplement: Table S4 — Peptides tested for binding the SH2 domains of GADS, GRB7 and HSH2D in the peptide array experiment. (DOC) [file pone.0025528.s006.doc]

**Table S4.** Peptides tested for binding the SH2 domains of GADS, GRB7 and HSH2D in the peptide array experiment.

| **Index** | **Peptide** | **Spot value GADS** | **Spot value GRB7** | **Spot value HSH2D** |
| --- | --- | --- | --- | --- |
| 1 | AHpYENLN | 126.28 | 130.26 | 26.63 |
| 2 | DSpYENMD | 128.02 | 127.42 | 22.95 |
| 3 | DKpYENSI | 22.16 | 6.88 | 0.16 |
| 4 | HGpYENPT | 13.98 | 26.92 | 0.6 |
| 5 | ASpYENEG | 119 | 140.62 | 80.86 |
| 6 | GGpYENQK | 98.44 | 140.86 | 105.14 |
| 7 | SVpYENCL | 0 | 138.94 | 0.46 |
| 8 | ARpYENGH | 0 | 136.78 | 0 |
| 9 | GQpYENFR | 119.98 | 136.07 | 83.63 |
| 10 | DPpYENYR | 118.11 | 137.12 | 109.65 |
| 11 | ALpYDNVP | 118.15 | 135.72 | 127.46 |
| 12 | ADpYMNLH | 114.86 | 134.92 | 117.48 |
| 13 | DCpYDNAN | 121.84 | 132.85 | 79.46 |
| 14 | AIpYDNPN | 122.62 | 131.15 | 148.76 |
| 15 | AQpYDNES | 119.21 | 127.98 | 110.16 |
| 16 | SDpYMNMT | 123.43 | 130.83 | 134.67 |
| 17 | AMpYQNVD | 43.04 | 119.4 | 3.37 |
| 18 | RPpYWNMT | 122.09 | 127.64 | 127.24 |
| 19 | ASpYVNLP | 126.73 | 125.51 | 136.78 |
| 20 | ARpYANVP | 125.04 | 128.35 | 140.98 |
| 21 | DHpYNNLS | 128.18 | 121.01 | 120.37 |
| 22 | AKpYLNMR | 127.72 | 101.45 | 109.64 |
| 23 | LKpYMNSG | 125.48 | 122.87 | 152.11 |
| 24 | EEpYWNSS | 100.38 | 117.35 | 100.36 |
| 25 | KLpYANMF | 120.68 | 116.21 | 115.39 |
| 26 | GQpYDNDA | 120.26 | 117.96 | 71.36 |
| 27 | ADpYGNLM | 126.19 | 121.63 | 120.08 |
| 28 | KEpYTNWE | 62.65 | 107.56 | 43.94 |
| 29 | YTpYNNWS | 124.39 | 126.95 | 149.2 |
| 30 | EDpYQNSA | 0 | 102.38 | 107.79 |
| 31 | RRpYQNPS | 124.75 | 125.41 | 146.37 |
| 32 | HVpYQNEN | 0 | 117.12 | 100.05 |
| 33 | FSpYVNPQ | 126.38 | 127.55 | 119.09 |
| 34 | TVpYLNEK | 125.47 | 127.01 | 137.21 |
| 35 | AEpYTNSP | 126.46 | 128.17 | 135.73 |
| 36 | DEpYSNPA | 108.94 | 118.74 | 127.75 |
| 37 | QPpYANEG | 127.69 | 117.05 | 150.92 |
| 38 | ELpYNNST | 126.95 | 128.46 | 20.34 |
| 39 | NQpYNNPA | 125.89 | 129.68 | 114.33 |
| 40 | HLpYNNEE | 47.23 | 124.06 | 48.58 |
| 41 | DGpYDNRL | 127 | 126.82 | 69.51 |
| 42 | DLpYCNTR | 126.77 | 53.33 | 47.26 |
| 43 | EVpYCNPK | 127.54 | 129.95 | 50.23 |
| 44 | TPpYMNGE | 127.44 | 129.79 | 91.89 |
| 45 | NEpYPNIT | 112.19 | 101.58 | 134.93 |
| 46 | ALpYKNLL | 130.47 | 142.97 | 151.8 |
| 47 | AApYVNNL | 133.91 | 129.88 | 27.82 |
| 48 | DApYDNFD | 134.37 | 140.85 | 140.66 |
| 49 | APpYDNYV | 132.74 | 112.69 | 79.9 |
| 50 | GIpYANQE | 94.05 | 140.67 | 112.68 |
| 51 | PApYGNSP | 104.59 | 139.42 | 122.75 |
| 52 | PSpYGNPS | 124.28 | 137.44 | 18.66 |
| 53 | DLpYANNV | 117.89 | 113.18 | 1.08 |
| 54 | QPpYNNQG | 126.11 | 125.09 | 7.12 |
| 55 | AIpYGNEP | 115.28 | 134.05 | 76.84 |
| 56 | GQpYLNCI | 120.49 | 135.76 | 139.82 |
| 57 | VLpYHNMK | 120.54 | 122.79 | 21.22 |
| 58 | WPpYQNGF | 131.12 | 110.14 | 0.15 |
| 59 | GEpYFNVP | 128.94 | 87.13 | 32.6 |
| 60 | GEpYYNVP | 127.84 | 120.52 | 40.36 |
| 61 | GIpYINGR | 129.55 | 126.03 | 76.09 |
| 62 | YDpYLNDW | 127.72 | 116.92 | 125.14 |
| 63 | YRpYPNWS | 133.94 | 90.63 | 12.39 |
| 64 | DMpYTNGS | 130.48 | 52.42 | 8.39 |
| 65 | FIpYGNQD | 134.18 | 115.25 | 107.17 |
| 66 | AApYMNKV | 131.83 | 73.09 | 115.2 |
| 67 | DDpYSNDP | 118.29 | 128.4 | 100.45 |
| 68 | ITpYGNNK | 130.24 | 94.21 | 95.42 |
| 69 | NCpYFNMK | 120.98 | 117.01 | 137.73 |
| 70 | SDpYYNML | 134.56 | 108.74 | 33.47 |
| 71 | PDpYWNHS | 131.66 | 104.13 | 41.48 |
| 72 | RNpYPNTF | 126.11 | 128.25 | 56.83 |
| 73 | ELpYRNAY | 125.3 | 124.13 | 129.16 |
| 74 | DDpYHNPG | 132.65 | 119.41 | 7.94 |
| 75 | CEpYPNED | 132.88 | 66.97 | 13.04 |
| 76 | AMpYRNEG | 28.78 | 118.22 | 6.12 |
| 77 | VKpYMNFY | 131.39 | 124.59 | 128.56 |
| 78 | HTpYQNRR | 133.58 | 96.89 | 25.22 |
| 79 | MIpYGNGA | 132.38 | 121.84 | 27.14 |
| 80 | ARpYLNRN | 133.31 | 116.82 | 99.69 |
| 81 | PYpYGNDS | 129.87 | 129.67 | 152.32 |
| 82 | ASpYFNSK | 133.14 | 118.83 | 13.48 |
| 83 | DYpYYNTD | 133.49 | 125.3 | 73.92 |
| 84 | FGpYSNRV | 132.66 | 131.17 | 2.43 |
| 85 | KFpYYNPN | 132.85 | 136.3 | 137.14 |
| 86 | GApYYNEA | 134.57 | 133.75 | 76.78 |
| 87 | GQpYNNHL | 70.02 | 6.54 | 0.42 |
| 88 | EWpYQNFQ | 133.26 | 101.23 | 85.86 |
| 89 | LLpYQNYR | 120.58 | 136.23 | 136.43 |
| 90 | PVpYHNQP | 117.72 | 138.04 | 133.62 |
| 91 | DNpYLNFS | 128.4 | 29.63 | 15.78 |
| 92 | DIpYVNYP | 138.32 | 128.37 | 153.34 |
| 93 | KApYTNFD | 139.68 | 143.13 | 136.76 |
| 94 | DDpYANYN | 134.01 | 131.74 | 88.19 |
| 95 | SLpYHNCL | 132.53 | 128.06 | 18.39 |
| 96 | AQpYNNFS | 132.29 | 135.05 | 134.88 |
| 97 | MRpYNNYK | 129.29 | 124.16 | 55.1 |
| 98 | LTpYGNRY | 72.45 | 84.55 | 14.19 |
| 99 | IDpYFNNQ | 120.59 | 73.12 | 11.59 |
| 100 | MGpYYNNS | 122.07 | 114.45 | 17.06 |
| 101 | PRpYPNDS | 122.95 | 123 | 78.18 |
| 102 | DIpYKNDY | 120.75 | 114.38 | 4.53 |
| 103 | DCpYGNYD | 94.02 | 71.03 | 25.31 |
| 104 | YApYFNGC | 124.92 | 111.78 | 147.01 |
| 105 | LGpYYNGK | 135.89 | 75.57 | 8.37 |
| 106 | HQpYYNDF | 136.4 | 97.17 | 6.18 |
| 107 | GKpYPNRG | 135.89 | 121.09 | 132.21 |
| 108 | DPpYKNRV | 115.91 | 55.16 | 75.49 |
| 109 | PRpYPNYM | 66.85 | 54.38 | 0.15 |
| 110 | DEpYKNFY | 20.42 | 3.72 | 2.2 |
| 111 | EPpYHNYR | 119.21 | 124.61 | 135.4 |
| 112 | RRpYFNKP | 138.15 | 124.11 | 144.11 |
| 113 | LKpYYNKP | 135.75 | 122.01 | 48.36 |
| 114 | DDpYFNYN | 117.13 | 121.3 | 67.44 |
| 115 | PFpYEMLA | 125.25 | 106.85 | 94.84 |
| 116 | DGpYEQLR | 129.38 | 128.07 | 103.49 |
| 117 | ADpYEEIL | 126.64 | 130.79 | 105.85 |
| 118 | CGpYEGLG | 111.24 | 83.92 | 68.16 |
| 119 | EApYEMPS | 123.7 | 94.78 | 1.73 |
| 120 | DPpYEQWS | 66.09 | 61.99 | 0 |
| 121 | LApYEPVW | 126.83 | 116.4 | 99.88 |
| 122 | VTpYEWAP | 61.87 | 73.27 | 4.07 |
| 123 | CPpYEQAQ | 135.44 | 120.99 | 71.53 |
| 124 | CTpYEAMY | 43.31 | 7.82 | 0.13 |
| 125 | EKpYECAL | 135.56 | 135.05 | 107.36 |
| 126 | QLpYEWQQ | 136.4 | 132.35 | 73.47 |
| 127 | YDpYEAWA | 136.58 | 120.83 | 113.59 |
| 128 | AVpYEEPP | 136.47 | 84.25 | 29.32 |
| 129 | ENpYEGSE | 138.14 | 65.08 | 1.49 |
| 130 | RQpYEQQT | 136.86 | 67.23 | 1.21 |
| 131 | GLpYELWK | 136.3 | 59.78 | 3.35 |
| 132 | ALpYETPT | 140.14 | 137.69 | 128.88 |
| 133 | DGpYETEG | 138.33 | 141.81 | 21.25 |
| 134 | ADpYEPPP | 137.79 | 102.25 | 2.52 |
| 135 | NPpYEPEL | 137.67 | 100.62 | 10.23 |
| 136 | TPpYEWDL | 34.82 | 115.06 | 5.66 |
| 137 | DSpYELTG | 129.63 | 118.15 | 150.63 |
| 138 | DIpYERMV | 130.69 | 121.64 | 44.86 |
| 139 | AEpYEFLS | 141.97 | 135.67 | 147.12 |
| 140 | DFpYELEP | 143.8 | 134.18 | 31.8 |
| 141 | DDpYEDMM | 125.37 | 106.78 | 135.33 |
| 142 | KCpYECGK | 119.97 | 43.8 | 32.07 |
| 143 | AGpYEAQG | 107.66 | 117.58 | 140.8 |
| 144 | DGpYECDV | 120.36 | 110.51 | 127.19 |
| 145 | WLpYEEGE | 1.6 | 2.13 | 0.03 |
| 146 | KDpYEFMW | 108.01 | 91.26 | 53.99 |
| 147 | AKpYEGGP | 117.7 | 91.06 | 44.5 |
| 148 | DVpYEEDP | 130.39 | 105.53 | 63.3 |
| 149 | FLpYEPCG | 132.93 | 112.9 | 105.07 |
| 150 | ALpYEKAD | 69.51 | 83.4 | 6.51 |
| 151 | NIpYEGDL | 109.92 | 104.17 | 117.91 |
| 152 | EDpYERER | 122.29 | 41.53 | 46.43 |
| 153 | EEpYEDSS | 130.23 | 97.09 | 3.91 |
| 154 | PIpYEPGL | 131.61 | 106.52 | 100.25 |
| 155 | DIpYETDY | 50.12 | 66.52 | 54.57 |
| 156 | GPpYELGM | 127.63 | 66.94 | 1.55 |
| 157 | FEpYEYAM | 135.73 | 100.99 | 76 |
| 158 | APpYEFPE | 113.43 | 20.58 | 53.9 |
| 159 | FIpYEFEH | 116.68 | 107.86 | 29.49 |
| 160 | DIpYEKQT | 135.3 | 93.85 | 132.9 |
| 161 | GEpYEQFE | 0 | 0 | 4.43 |
| 162 | ADpYEGKD | 134.01 | 126.81 | 145.7 |
| 163 | NIpYEDNG | 122.7 | 119.94 | 54.08 |
| 164 | PTpYEEFP | 106.07 | 100.01 | 34.85 |
| 165 | AEpYEARS | 98.79 | 128.8 | 100.77 |
| 166 | KApYEYQE | 119.26 | 105.4 | 44.42 |
| 167 | RDpYEYNR | 94.62 | 101.99 | 52.64 |
| 168 | EKpYEGYL | 124.06 | 128.91 | 4.63 |
| 169 | TQpYERDF | 113.82 | 130.58 | 144.81 |
| 170 | KIpYEDGD | 11.82 | 80.02 | 1.12 |
| 171 | DGpYESYG | 134.43 | 116.36 | 16.89 |
| 172 | ITpYEPFY | 137.38 | 87.04 | 1.82 |
| 173 | SQpYEVFR | 138.97 | 117.68 | 23.92 |
| 174 | GDpYEFDS | 126.37 | 47.03 | 17.04 |
| 175 | KDpYDWIN | 29.94 | 48.78 | 4.53 |
| 176 | DNpYDQLV | 134.7 | 80.11 | 27.49 |
| 177 | DIpYDCVP | 129.41 | 84.74 | 27.19 |
| 178 | AQpYEKYS | 132.55 | 124.46 | 99.49 |
| 179 | QKpYEYKS | 129.41 | 0.02 | 2.03 |
| 180 | DDpYEDFD | 129.6 | 127.11 | 107.21 |
| 181 | DApYDGVT | 141.36 | 134.61 | 14.95 |
| 182 | SLpYDMAR | 142.02 | 125.31 | 36.82 |
| 183 | KVpYEFYS | 137.81 | 127.44 | 133.81 |
| 184 | DLpYMWLS | 125.17 | 91.42 | 66.48 |
| 185 | QNpYDEMS | 138.35 | 61.16 | 30.42 |
| 186 | ANpYWWLR | 108.28 | 93.06 | 150.32 |
| 187 | ELpYMMMR | 138.83 | 78.5 | 96.55 |
| 188 | DPpYDQSF | 140.73 | 124.18 | 56.37 |
| 189 | LEpYDQEH | 141.2 | 96.8 | 121.02 |
| 190 | QVpYDAMG | 3.5 | 0.19 | 2.06 |
| 191 | AQpYSMLA | 4.21 | 26.68 | 17.36 |
| 192 | DYpYNMLL | 0 | 12.18 | 0 |
| 193 | ELpYDIMK | 98.47 | 14.48 | 0 |
| 194 | PNpYMGVG | 63.89 | 48.95 | 25.21 |
| 195 | AEpYQQLQ | 131.69 | 102.79 | 49.05 |
| 196 | LTpYDGAL | 126.02 | 78 | 44.88 |
| 197 | EVpYDGPK | 112.7 | 106.3 | 51.15 |
| 198 | FApYLMMR | 129.97 | 51.19 | 2.55 |
| 199 | SNpYDQQH | 0 | 0 | 0.6 |
| 200 | AApYDKLE | 56.49 | 92.11 | 139.09 |
| 201 | ALpYAQVK | 128.8 | 42.31 | 8.78 |
| 202 | AVpYSMMS | 36.28 | 0 | 0 |
| 203 | AQpYDAAH | 128.84 | 106.26 | 5.11 |
| 204 | HIpYMEMN | 120.62 | 94.42 | 91.24 |
| 205 | AQpYDTPK | 132.15 | 109.1 | 104.01 |
| 206 | AApYQELC | 0 | 0 | 1.18 |
| 207 | AQpYDDIV | 115.69 | 60.45 | 77.87 |
| 208 | DQpYLMWL | 82.72 | 106.73 | 124.38 |
| 209 | FVpYDCNA | 59.23 | 23.57 | 0.69 |
| 210 | YQpYWPVL | 64.52 | 43.3 | 0 |
| 211 | NHpYDPEE | 0 | 0 | 0 |
| 212 | EVpYQGVC | 70.14 | 108.33 | 26.27 |
| 213 | EHpYNCIS | 131.08 | 123.59 | 92.33 |
| 214 | HIpYDEQP | 59.55 | 118.3 | 72.6 |
| 215 | AVpYIGVH | 27.71 | 47.23 | 1.41 |
| 216 | RVpYMQSQ | 0 | 14.86 | 0.27 |
| 217 | AKpYGWVT | 125.56 | 132.65 | 124.89 |
| 218 | NDpYMQPE | 134.58 | 124.1 | 115.9 |
| 219 | EKpYDKMR | 92.72 | 126.59 | 18.14 |
| 220 | DLpYDGQV | 120.17 | 80.88 | 32.13 |
| 221 | ALpYDFLP | 122.58 | 133.93 | 16.23 |
| 222 | KPpYCCLV | 0.86 | 9.41 | 0 |
| 223 | AQpYMAME | 137.56 | 137.84 | 119.19 |
| 224 | LHpYDQGC | 101.98 | 58.84 | 1.63 |
| 225 | HApYAQML | 107.99 | 98.06 | 129.1 |
| 226 | DIpYVCMI | 90.09 | 107.1 | 111.61 |
| 227 | MDpYWQPP | 141.63 | 74.45 | 11.78 |
| 228 | DDpYMPMS | 142 | 133.23 | 115.67 |
| 229 | HIpYQPVG | 142.93 | 115.4 | 128.84 |
| 230 | VTpYNGVD | 121.07 | 95.93 | 141.7 |
| 231 | RDpYDDMS | 0 | 2.98 | 0 |
| 232 | ATpYLSVA | 128.51 | 121.68 | 137.6 |
| 233 | GDpYGQLH | 134.97 | 89.16 | 11.05 |
| 234 | PNpYDKWE | 106.88 | 107.24 | 142.25 |
| 235 | GLpYDSQN | 29.82 | 71.95 | 51.78 |
| 236 | DTpYVEMR | 129.16 | 109.75 | 55.54 |
| 237 | CVpYSMAD | 13.87 | 26.31 | 56.4 |
| 238 | GLpYCGVA | 134.24 | 74.87 | 4.49 |
| 239 | AQpYDSNS | 0 | 0 | 0 |
| 240 | LApYTMER | 29.6 | 0 | 0 |
| 241 | SQpYNMAG | 128.61 | 59.77 | 55.49 |
| 242 | ADpYATLY | 0 | 0 | 0 |
| 243 | QPpYDPNF | 3.54 | 0 | 0 |
| 244 | EApYGWMD | 126.39 | 0 | 0 |
| 245 | LGpYDEGS | 26.52 | 0 | 0 |
| 246 | AGpYSPIM | 62.7 | 59.23 | 0 |
| 247 | DApYGCLG | 128.36 | 120.6 | 114.29 |
| 248 | GDpYTWTE | 25.22 | 80.23 | 39.01 |
| 249 | GEpYWWNA | 23.45 | 60.05 | 9.93 |
| 250 | FSpYAWPF | 3.45 | 52.67 | 56.85 |
| 251 | SDpYDMHT | 0 | 0 | 0 |
| 252 | RCpYCMTD | 118.7 | 0 | 3.43 |
| 253 | AIpYNPVI | 0 | 0 | 0 |
| 254 | ADpYIEWL | 132.47 | 33.36 | 89.98 |
| 255 | MVpYDACR | 0 | 121.2 | 1.12 |
| 256 | LVpYSGMG | 0 | 126.43 | 1.01 |
| 257 | AGpYIQTG | 0 | 123.15 | 0 |
| 258 | GLpYDVQA | 0 | 126.91 | 0 |
| 259 | ATpYCTVC | 0 | 106.81 | 0 |
| 260 | QKpYMVWS | 0 | 130.82 | 2.49 |
| 261 | TMpYMQNT | 2.16 | 83.02 | 0 |
| 262 | IDpYDLNL | 0 | 98.21 | 0 |
| 263 | ALpYDKTK | 0 | 104.09 | 0 |
| 264 | IIpYCPVN | 0 | 126.62 | 0 |
| 265 | ELpYCEMG | 0.24 | 130.23 | 0 |
| 266 | FGpYDKPH | 0 | 74.03 | 0 |
| 267 | ISpYPMLL | 0 | 107.22 | 0 |
| 268 | DPpYWKIK | 6.95 | 135.02 | 0.16 |
| 269 | DGpYDRED | 0 | 130.55 | 0 |
| 270 | DHpYVMQE | 0 | 110.62 | 0 |
| 271 | AKpYNIIT | 0 | 127.58 | 0 |
| 272 | ARpYMAPE | 0 | 131.51 | 0 |
| 273 | KRpYWQNL | 0 | 77.43 | 1.46 |
| 274 | AEpYAQPL | 0 | 131.19 | 0 |
| 275 | VLpYMPSM | 3.96 | 37.31 | 0 |
| 276 | ERpYSQEV | 13.47 | 83.72 | 0 |
| 277 | FRpYVCEG | 3.43 | 73.85 | 1.52 |
| 278 | ERpYQEAA | 26.64 | 142.75 | 4.22 |
| 279 | APpYWTSP | 0 | 136.3 | 0 |
| 280 | DLpYNQPE | 8.11 | 136.79 | 11.87 |
| 281 | HSpYDDST | 1.29 | 132.22 | 2.38 |
| 282 | AIpYDICR | 0 | 92.44 | 1.06 |
| 283 | AIpYKWIT | 0 | 99.75 | 0.83 |
| 284 | TIpYDMYR | 2.03 | 113.08 | 0.35 |
| 285 | AVpYCIIG | 2.36 | 87.06 | 0 |
| 286 | KQpYQEEI | 0.89 | 109.13 | 0 |
| 287 | RIpYDPGG | 0 | 30.98 | 0 |
| 288 | TGpYWPTQ | 0 | 90.1 | 0 |
| 289 | HDpYDDER | 0.13 | 109.81 | 0 |
| 290 | KLpYGMSD | 0 | 20.03 | 0 |
| 291 | EPpYIAWG | 0 | 9.88 | 0 |
| 292 | EApYDQRQ | 0 | 91.83 | 0 |
| 293 | ELpYMWDS | 0 | 57.15 | 0 |
| 294 | KGpYVWNG | 0 | 125.29 | 0 |
| 295 | TGpYGMPR | 0 | 84.24 | 0 |
| 296 | DQpYVLMS | 0 | 42.89 | 0 |
| 297 | DApYWPEA | 0 | 59.51 | 4.78 |
| 298 | TDpYMENG | 0 | 29.66 | 0 |
| 299 | IDpYMISD | 0 | 121.02 | 0 |
| 300 | AKpYGSLR | 0 | 44.74 | 0 |
| 301 | APpYDWFG | 0 | 102.6 | 0 |
| 302 | DQpYMKMT | 0 | 64.16 | 0 |
| 303 | INpYNCEI | 0 | 82.91 | 0 |
| 304 | PEpYMLEK | 0 | 108.15 | 0 |
| 305 | GPpYLGPP | 0 | 112.85 | 0 |
| 306 | ETpYGEMA | 0 | 108.97 | 0.27 |
| 307 | GEpYSEAI | 0 | 101.57 | 0.37 |
| 308 | DKpYIGES | 0 | 66.28 | 0.2 |
| 309 | KEpYCPMV | 0 | 101.38 | 0 |
| 310 | AApYDFSQ | 76.77 | 135.46 | 0.52 |
| 311 | AIpYTIMR | 0 | 93.48 | 0 |
| 312 | DWpYDCHR | 0 | 114.65 | 0 |
| 313 | HIpYPQLS | 0 | 98.15 | 0 |
| 314 | CNpYKQLR | 0 | 88.22 | 0 |
| 315 | SQpYPMMR | 0 | 115.69 | 0.38 |
| 316 | LCpYWVPE | 0 | 127.65 | 0 |
| 317 | ELpYDYPK | 0 | 107.88 | 0 |
| 318 | DGpYSEEE | 0 | 136.65 | 0.06 |
| 319 | SPpYCCPE | 0 | 136.52 | 1.32 |
| 320 | CSpYTMCP | 0 | 137.06 | 1.34 |
| 321 | PNpYNWNS | 0 | 86.12 | 0 |
| 322 | NSpYFMVE | 7.56 | 137.69 | 0.94 |
| 323 | AKpYWLER | 0 | 136.31 | 0.15 |
| 324 | EDpYDYES | 0 | 119.69 | 0 |
| 325 | FGpYMECS | 0 | 140.46 | 3.11 |
| 326 | GGpYMDMS | 0 | 108.02 | 0 |
| 327 | LNpYQTPG | 0.47 | 14.32 | 0.02 |
| 328 | EEpYDLDA | 0.06 | 24.21 | 0 |
| 329 | NCpYDRQE | 0 | 28.22 | 0.23 |
| 330 | ESpYQTEA | 0.35 | 65.11 | 0.52 |
| 331 | AHpYQPTG | 0 | 24.71 | 0 |
| 332 | DLpYNGAT | 0 | 81.93 | 0 |
| 333 | EQpYDEKR | 0.16 | 121.85 | 0 |
| 334 | IRpYMTQG | 0 | 49.86 | 0 |
| 335 | AApYLTSL | 0 | 77.77 | 0 |
| 336 | DYpYDRNL | 1.08 | 130.11 | 0 |
| 337 | AIpYKCLL | 0 | 119.6 | 0 |
| 338 | DPpYSQQI | 0 | 27.91 | 0 |
| 339 | AEpYLTPV | 0 | 35.05 | 0 |
| 340 | DGpYYWIT | 0 | 84.98 | 0 |
| 341 | QTpYGEWN | 0 | 113.45 | 0 |
| 342 | AYpYGQTP | 6.64 | 111.24 | 0 |
| 343 | AQpYLSEI | 0 | 117.77 | 0 |
| 344 | ASpYMPQF | 16.79 | 113.07 | 0 |
| 345 | GLpYLMDG | 0 | 50.56 | 0 |
| 346 | GLpYGQPG | 0 | 115.99 | 0 |
| 347 | DLpYIPPP | 0 | 83.09 | 0 |
| 348 | DVpYDGRF | 0 | 77.71 | 0 |
| 349 | QRpYQEQG | 0 | 81.82 | 0 |
| 350 | DMpYGQEK | 0 | 27.08 | 0 |
| 351 | YGpYWGCA | 0 | 38.8 | 0 |
| 352 | SKpYCGPY | 0 | 53.96 | 0 |
| 353 | AEpYGGWY | 0 | 60.54 | 0 |
| 354 | ETpYQENT | 4.02 | 59.59 | 1.11 |
| 355 | AApYQIAI | 0 | 98.8 | 0 |
| 356 | SDpYMFMA | 0 | 122.05 | 0 |
| 357 | GGpYTCQS | 0 | 45.46 | 0 |
| 358 | LEpYMMKC | 0 | 134.51 | 0 |
| 359 | EEpYLEQS | 0 | 73.21 | 0 |
| 360 | HYpYDKCP | 0 | 109.59 | 0 |
| 361 | ANpYSAEQ | 0 | 89.79 | 0 |
| 362 | DPpYQYVV | 0 | 123.35 | 0 |
| 363 | ASpYTPTP | 0 | 133.91 | 0 |
| 364 | LLpYAMDS | 0 | 97.4 | 0 |
| 365 | GDpYCRIN | 0 | 97.28 | 0 |
| 366 | DDpYSPPS | 0 | 135.72 | 0 |
| 367 | LRpYGMNP | 0 | 128.05 | 0 |
| 368 | SRpYMEDS | 0 | 84.63 | 0 |
| 369 | PApYQQGQ | 0 | 95.98 | 0 |
| 370 | LHpYMVQT | 0 | 106.89 | 0 |
| 371 | FVpYNSER | 0 | 121.68 | 0.05 |
| 372 | AQpYPGIL | 0.56 | 132.11 | 0.52 |
| 373 | ILpYSQCG | 0 | 112.44 | 0 |
| 374 | EVpYKGID | 1 | 131.34 | 1.55 |
| 375 | GGpYYQIT | 1.01 | 97.11 | 0.12 |
| 376 | DPpYVRMY | 1.39 | 127.74 | 0 |
| 377 | GPpYVGQA | 0.76 | 15.04 | 0 |
| 378 | ERpYLFVD | 0.78 | 0 | 0.03 |
| 379 | EYpYNWGR | 0.44 | 38.72 | 0 |
| 380 | AYpYVLEG | 0.56 | 51.45 | 0 |
| 381 | STpYDEYE | 0.37 | 87.41 | 0 |
| 382 | DDpYMKSL | 1.44 | 109.41 | 0 |
| 383 | EEpYTEQS | 1.88 | 120.85 | 0 |
| 384 | GGpYLQGN | 0 | 85.6 | 0 |
| 385 | QYpYLGNF | 0 | 76.67 | 0 |
| 386 | KApYAWDT | 0.66 | 26.88 | 0.18 |
| 387 | ALpYDYQE | 0.01 | 82.57 | 0 |
| 388 | PDpYQQDF | 2.25 | 121.62 | 0 |
| 389 | ELpYGTWR | 0.53 | 102.4 | 0 |
| 390 | KEpYGWNL | 0 | 127.02 | 0 |
| 391 | RTpYGEPE | 0 | 66.59 | 0 |
| 392 | EQpYNEQY | 0 | 50.11 | 0 |
| 393 | AFpYSLSP | 0 | 82.87 | 0 |
| 394 | KPpYQCGQ | 0 | 88.33 | 0 |
| 395 | RYpYPMAG | 0 | 98.1 | 0 |
| 396 | ELpYDRGN | 0 | 41.37 | 0 |
| 397 | AQpYQKWD | 0 | 73.69 | 0 |
| 398 | AKpYAVPD | 0 | 60.57 | 0 |
| 399 | DSpYWHSR | 0 | 13.19 | 0 |
| 400 | PRpYGMCP | 0 | 80.55 | 0 |
| 401 | PDpYLECL | 0 | 78.55 | 0 |
| 402 | DQpYYCVD | 0 | 2.03 | 0 |
| 403 | DMpYAYLL | 0 | 126.88 | 0.75 |
| 404 | GKpYPMPN | 0 | 79.1 | 0 |
| 405 | LPpYHMPE | 0 | 84.16 | 0 |
| 406 | GEpYCPEV | 0 | 97.63 | 0 |
| 407 | ETpYVQDR | 0 | 42.09 | 0 |
| 408 | CIpYGGAP | 0 | 57.66 | 0 |
| 409 | VNpYPMEN | 0 | 65 | 0 |
| 410 | IKpYNCCE | 0 | 60.02 | 0 |
| 411 | EKpYNVPL | 5.31 | 126.16 | 0 |
| 412 | GQpYQPQP | 0 | 76.3 | 0 |
| 413 | AKpYPSLG | 0 | 118.84 | 0 |
| 414 | GGpYSGNS | 0 | 89.3 | 0 |
| 415 | DLpYNYIN | 0 | 114.51 | 0 |
| 416 | KKpYNVEY | 0 | 82.15 | 0 |
| 417 | DLpYGGEK | 0 | 121.88 | 0 |
| 418 | DTpYCEQK | 0 | 96.85 | 0 |
| 419 | GIpYNGNI | 10.96 | 28.53 | 0.36 |
| 420 | VVpYPWTQ | 0 | 108.62 | 0.35 |
| 421 | IPpYKWTA | 0 | 56.69 | 0 |
| 422 | PTpYGQQG | 0.98 | 137.17 | 0.85 |
| 423 | EDpYMSDR | 2.63 | 73.58 | 0 |
| 424 | DIpYLANI | 1.66 | 12.27 | 0.26 |
| 425 | DVpYDDGK | 1.9 | 2.64 | 0 |
| 426 | ADpYIPQL | 2 | 20.37 | 0 |
| 427 | IHpYWDTT | 2.02 | 103.6 | 0.83 |
| 428 | AEpYFELP | 1.26 | 25.77 | 0 |
| 429 | PEpYQYMP | 2.59 | 102.62 | 0 |
| 430 | NRpYWLCA | 5.78 | 113.26 | 0 |
| 431 | SNpYCLPS | 2.09 | 105.84 | 0 |
| 432 | SIpYGIWF | 1.95 | 64.89 | 0 |
| 433 | AVpYGQND | 2.22 | 95.44 | 0 |
| 434 | VVpYLPND | 0.9 | 90.92 | 0 |
| 435 | DApYDSYW | 0.95 | 91.48 | 0 |
| 436 | GApYGMGG | 0 | 91.57 | 0 |
| 437 | ARpYARWA | 0.41 | 54.81 | 0 |
| 438 | AIpYTTQM | 0.25 | 101.76 | 0 |
| 439 | QIpYQEDE | 0 | 38.79 | 0 |
| 440 | RLpYWADA | 0 | 113.92 | 0 |
| 441 | LApYQLQV | 0 | 10.38 | 0 |
| 442 | AApYFGIY | 0 | 0 | 0 |
| 443 | EEpYIYMN | 1.01 | 50.54 | 0 |
| 444 | SDpYDPFI | 0 | 61.66 | 0 |
| 445 | AKpYVMKT | 0 | 65.71 | 0 |
| 446 | ALpYSSNL | 0 | 0 | 0 |
| 447 | AFpYAPQK | 0 | 84.57 | 0 |
| 448 | ELpYMCHK | 0 | 34.57 | 0 |
| 449 | AQpYQVNQ | 0 | 69.96 | 0 |
| 450 | EDpYGDIE | 0 | 30.81 | 0 |
| 451 | GEpYGAEA | 0 | 39.39 | 0 |
| 452 | EQpYQKTE | 0 | 75.43 | 0 |
| 453 | MApYGPAQ | 0 | 73.5 | 0 |
| 454 | ADpYLSCD | 0 | 96.62 | 0 |
| 455 | DDpYQGDS | 85.3 | 74.13 | 1.27 |
| 456 | GApYQKPT | 0 | 70.44 | 0 |
| 457 | ETpYSPNT | 0 | 95.42 | 0 |
| 458 | PKpYREWH | 0 | 51.89 | 0 |
| 459 | GHpYTEGA | 0.74 | 118.8 | 0 |
| 460 | SDpYGPEP | 30.69 | 121.63 | 0 |
| 461 | GLpYDFGP | 0 | 132.08 | 0 |
| 462 | PVpYVPCV | 0 | 67.14 | 0 |
| 463 | DYpYVLNG | 0 | 118.53 | 0.07 |
| 464 | EKpYSFMA | 0 | 138.58 | 0 |
| 465 | ATpYNPNM | 0 | 107.64 | 0 |
| 466 | EVpYFMAI | 0.14 | 34.01 | 0 |
| 467 | DApYAMKR | 0.45 | 82.73 | 0 |
| 468 | GRpYPQEN | 0.3 | 65.42 | 0 |
| 469 | AGpYVGDR | 0.41 | 54.04 | 0 |
| 470 | FDpYYMPA | 1.39 | 58.31 | 2.51 |
| 471 | LYpYMEKE | 2.62 | 64.79 | 0.08 |
| 472 | VIpYHGWT | 2.32 | 101.58 | 0.49 |
| 473 | QIpYDIFQ | 2.74 | 0.39 | 0.07 |
| 474 | EEpYMKNL | 2.62 | 91.55 | 0.08 |
| 475 | VLpYYMEK | 1.93 | 38.13 | 0.09 |
| 476 | AVpYAACQ | 2.69 | 5.36 | 0 |
| 477 | NWpYNMRN | 4.87 | 41.41 | 0 |
| 478 | DGpYSEDE | 2.53 | 42.63 | 0 |
| 479 | DRpYFAIT | 1.89 | 49.96 | 0 |
| 480 | DGpYDYDG | 1.87 | 44.56 | 0 |
| 481 | PHpYQDPH | 1.59 | 5.88 | 0 |
| 482 | ALpYKMQG | 3.4 | 95.56 | 0 |
| 483 | DTpYHPMS | 0.76 | 1.33 | 0 |
| 484 | PSpYNGGE | 0.56 | 43.01 | 0 |
| 485 | DDpYQDED | 0.92 | 102.28 | 0 |
| 486 | AYpYAPCG | 0.88 | 28.83 | 0 |
| 487 | DEpYGLPS | 0.3 | 86.21 | 0 |
| 488 | GVpYNVQY | 0.09 | 67.79 | 0 |
| 489 | YGpYGGQK | 0.43 | 49.52 | 0 |
| 490 | VMpYYEMS | 1.25 | 15.11 | 0 |
| 491 | GLpYGFLN | 0.32 | 27.23 | 0 |
| 492 | AHpYTHSD | 0.99 | 96.37 | 0 |
| 493 | ERpYLSGK | 0.06 | 0 | 0 |
| 494 | VIpYKCPK | 0 | 17.77 | 0 |
| 495 | SQpYKMNI | 0.18 | 11.48 | 0 |
| 496 | EPpYGLED | 0.24 | 10.65 | 0.18 |
| 497 | ATpYMDQA | 0.55 | 38.99 | 0 |
| 498 | ARpYAGDI | 0.66 | 131.96 | 0 |
| 499 | STpYYWPR | 0 | 2.55 | 0 |
| 500 | FDpYILCM | 0 | 0 | 0.01 |
| 501 | DEpYNPCQ | 1 | 32.14 | 0 |
| 502 | EApYIDPI | 0.56 | 39.1 | 0 |
| 503 | SGpYYWEV | 0.43 | 69.99 | 0 |
| 504 | LSpYNRAN | 0.48 | 11.81 | 0 |
| 505 | IEpYVDET | 0 | 20.19 | 0 |
| 506 | RSpYTYWW | 0.91 | 125.75 | 0 |
| 507 | KYpYNKPL | 0.46 | 94.56 | 0 |
| 508 | LDpYCGGS | 0.48 | 50.74 | 0 |
| 509 | ALpYIADR | 0.91 | 66.96 | 0 |
| 510 | LYpYWDQD | 0.32 | 37.47 | 0 |
| 511 | GTpYHAWK | 1.01 | 29.28 | 0.3 |
| 512 | LCpYGDMD | 1.05 | 142.31 | 0.31 |
| 513 | EMpYRLML | 0.7 | 29.66 | 0.06 |
| 514 | NGpYREPP | 1.08 | 43.93 | 0 |
| 515 | RIpYQFTA | 1.47 | 15.82 | 0.03 |
| 516 | KPpYQCKT | 13.43 | 54.7 | 1.37 |
| 517 | SVpYVPDE | 1.89 | 2.95 | 0.51 |
| 518 | PDpYAMYS | 65.82 | 136.88 | 20.08 |
| 519 | EPpYYLLL | 2.97 | 0 | 0.54 |
| 520 | ADpYSDPS | 2.89 | 68.55 | 1.54 |
| 521 | IKpYHEES | 2.65 | 18.42 | 0.48 |
| 522 | DNpYSPGW | 2.47 | 67.94 | 0.96 |
| 523 | ATpYCKPH | 2.22 | 16.29 | 0.46 |
| 524 | AGpYAERE | 2.69 | 0 | 0 |
| 525 | DVpYNDST | 2.65 | 36.94 | 0 |
| 526 | AApYMSKV | 2.68 | 32.75 | 0.18 |
| 527 | AFpYKGTV | 2.28 | 100.38 | 0 |
| 528 | AHpYNMYP | 1.86 | 0 | 0.19 |
| 529 | DTpYYQSP | 2.17 | 62.36 | 0.18 |
| 530 | ASpYSSDS | 1.04 | 35.45 | 0 |
| 531 | AGpYVIGM | 1.17 | 0 | 0 |
| 532 | RCpYMYQA | 0.9 | 126.47 | 1.08 |
| 533 | MKpYLYAY | 1.76 | 93 | 0 |
| 534 | ASpYNPGS | 0.88 | 0 | 0 |
| 535 | MQpYFQPT | 0.83 | 18.12 | 8.75 |
| 536 | VQpYQRQA | 0.89 | 24.43 | 0.39 |
| 537 | KFpYMPKK | 1.25 | 90.83 | 0 |
| 538 | GFpYLFPD | 0.75 | 21.67 | 0 |
| 539 | GEpYYQES | 0.42 | 56.97 | 0.18 |
| 540 | ASpYAPDA | 0.86 | 35.11 | 0.35 |
| 541 | EEpYVYEF | 8.61 | 110.72 | 1.09 |
| 542 | GEpYAWYY | 1.06 | 0 | 0.6 |
| 543 | SNpYFSMD | 3.71 | 52.42 | 0.31 |
| 544 | CIpYWTKY | 0.9 | 19 | 0.63 |
| 545 | EKpYQQYK | 0.49 | 0 | 0 |
| 546 | ITpYDDRF | 0.32 | 51.83 | 0.16 |
| 547 | AIpYGPNT | 0 | 0 | 0.06 |
| 548 | CDpYCDTY | 0.17 | 0 | 0 |
| 549 | PKpYGEGH | 6.33 | 127.19 | 0.38 |
| 550 | DLpYCDPR | 0.41 | 17.12 | 0.17 |
| 551 | GHpYPVWS | 0.54 | 74.24 | 0 |
| 552 | QRpYYPMA | 0.49 | 26.67 | 0 |
| 553 | NMpYPQNP | 0.18 | 0.8 | 0.03 |
| 554 | EFpYGYMT | 0.23 | 49.67 | 0.04 |
| 555 | AApYSVGE | 0.68 | 30.21 | 0 |
| 556 | MSpYYCSG | 0.92 | 73.87 | 0.67 |
| 557 | CRpYTYSE | 1.3 | 92.6 | 0.5 |
| 558 | CIpYQGRL | 2.21 | 142.93 | 0.68 |
| 559 | CSpYLQYL | 1.18 | 5.29 | 0.84 |
| 560 | AKpYMKDL | 1.6 | 132.02 | 0.68 |
| 561 | DGpYSYPS | 1.89 | 108.3 | 0.22 |
| 562 | PNpYFMNE | 2.35 | 136.13 | 1.07 |
| 563 | LYpYQDQL | 4.18 | 118.35 | 1.34 |
| 564 | GPpYPAAP | 2.18 | 42.68 | 0.23 |
| 565 | NApYYCEK | 2.33 | 43.8 | 0.68 |
| 566 | RKpYSFEC | 2.2 | 3.46 | 0.93 |
| 567 | ATpYNFSQ | 3.26 | 2.73 | 1.37 |
| 568 | EDpYGTCS | 2.52 | 7.63 | 0.89 |
| 569 | GQpYGIQK | 3.44 | 43.9 | 1.45 |
| 570 | AIpYPTPV | 2.27 | 34.03 | 0.44 |
| 571 | TApYQCFS | 2.49 | 44.53 | 0.34 |
| 572 | HPpYNFPE | 3.57 | 60.58 | 0.15 |
| 573 | AVpYDRYS | 2.62 | 3.18 | 0.26 |
| 574 | DApYMFCI | 2.21 | 6.86 | 0.17 |
| 575 | CDpYSIDG | 2.25 | 33.35 | 0.52 |
| 576 | EKpYKDLD | 15.07 | 72.06 | 0.7 |
| 577 | ADpYKAEQ | 1.86 | 57.13 | 0 |
| 578 | DRpYSRQG | 1.31 | 0 | 0 |
| 579 | HApYHPAC | 2.14 | 6.47 | 0 |
| 580 | DIpYGINQ | 1.25 | 1.95 | 0.25 |
| 581 | EIpYMTFL | 0.57 | 28.42 | 0 |
| 582 | FYpYRPPR | 2.53 | 64.09 | 0.53 |
| 583 | ESpYFETN | 0.71 | 13.54 | 0.45 |
| 584 | STpYFAWC | 1.48 | 50.92 | 0 |
| 585 | NPpYSQFQ | 0.87 | 0 | 0.22 |
| 586 | GGpYGGDR | 0.88 | 57.72 | 0.64 |
| 587 | DLpYSRNA | 0 | 41.83 | 1.15 |
| 588 | DHpYFVMT | 1.61 | 10.09 | 1.15 |
| 589 | IApYWLHP | 1.1 | 0 | 0.75 |
| 590 | NVpYCIGQ | 0.47 | 7.81 | 0.7 |
| 591 | LNpYCFSG | 0.53 | 16.79 | 1.08 |
| 592 | ALpYGKEK | 0.3 | 0 | 0.71 |
| 593 | ELpYYEEA | 1.13 | 7.01 | 0.55 |
| 594 | ETpYQEFR | 3.24 | 95.28 | 1.18 |
| 595 | LNpYPEQK | 1.6 | 30.85 | 0.86 |
| 596 | AApYQARQ | 2 | 72.3 | 1.43 |
| 597 | EPpYFGSL | 0.61 | 28.57 | 0.5 |
| 598 | GGpYDDYG | 1.22 | 91.36 | 0.65 |
| 599 | SSpYTDQF | 2.19 | 43.88 | 2.98 |
| 600 | FGpYYGPL | 1.23 | 7.29 | 0.41 |
| 601 | SFpYQFQH | 1.01 | 129.98 | 0.57 |
| 602 | NVpYCLDR | 1.46 | 31.86 | 0.71 |
| 603 | KYpYKENN | 1.32 | 98.41 | 0.89 |
| 604 | GSpYFGEI | 4.62 | 87.18 | 2.32 |
| 605 | DLpYQPHR | 2.95 | 45.32 | 0.84 |
| 606 | GDpYNGHV | 2.13 | 23.17 | 0.45 |
| 607 | SPpYACFY | 2.35 | 14.94 | 0.57 |
| 608 | LNpYPLPD | 2.59 | 1.88 | 1 |
| 609 | QQpYPRMT | 2.54 | 4.8 | 1.14 |
| 610 | GIpYQGFE | 2.9 | 2.56 | 1.42 |
| 611 | ARpYLTRG | 2.01 | 5.43 | 1.37 |
| 612 | CMpYPFIA | 3.07 | 32.83 | 2.24 |
| 613 | SYpYKGQT | 1.84 | 11.24 | 1.11 |
| 614 | APpYKFLS | 5.59 | 104.53 | 2.2 |
| 615 | VTpYCKNK | 1.59 | 84.64 | 1.14 |
| 616 | ANpYHLEN | 1.75 | 1.85 | 0.94 |
| 617 | ADpYYRVQ | 1.63 | 12.36 | 1.09 |
| 618 | GNpYVFQR | 2.56 | 28.13 | 1.59 |
| 619 | AVpYGQKE | 2.44 | 70.12 | 0.62 |
| 620 | LNpYMVYM | 1.35 | 28.04 | 0.47 |
| 621 | ITpYPQGL | 1.15 | 2.12 | 0.68 |
| 622 | AKpYHGNV | 0.74 | 3.86 | 0.83 |
| 623 | FVpYFQNH | 1.71 | 0 | 0.84 |
| 624 | DNpYWFGR | 2.9 | 0 | 0.61 |
| 625 | DDpYVGYS | 1.13 | 6.16 | 0 |
| 626 | AIpYQKDE | 0.77 | 8.34 | 0.82 |
| 627 | ERpYTEFY | 1.22 | 14.59 | 0.75 |
| 628 | DSpYFTSS | 1.74 | 37.21 | 0.99 |
| 629 | AApYAKKG | 4.01 | 110.54 | 0.91 |
| 630 | NGpYDFYP | 0.84 | 3.2 | 1.07 |
| 631 | DLpYQVRT | 31.54 | 90.66 | 1.34 |
| 632 | PGpYPQDL | 0.82 | 0 | 0.73 |
| 633 | EHpYFSPV | 1.12 | 68.59 | 1.66 |
| 634 | ERpYKQDV | 0 | 0 | 1.64 |
| 635 | RQpYADCS | 0.99 | 0 | 1.39 |
| 636 | FSpYAYQK | 0.8 | 0 | 1.43 |
| 637 | AHpYFDLS | 0.45 | 29.38 | 1.84 |
| 638 | ELpYNEFP | 1.23 | 8.85 | 1.34 |
| 639 | DEpYFSEQ | 0.78 | 63.94 | 1.12 |
| 640 | GTpYGLGD | 1.19 | 0 | 1.35 |
| 641 | QQpYFPSN | 8.93 | 140.12 | 2.65 |
| 642 | EDpYIRDW | 2.09 | 5.06 | 1.31 |
| 643 | DDpYGFSG | 33.7 | 118.2 | 0.66 |
| 644 | NSpYFMDV | 1.9 | 35.51 | 1.68 |
| 645 | LIpYKCGG | 1.39 | 29.04 | 1.18 |
| 646 | YSpYPKWF | 1.91 | 21 | 1.03 |
| 647 | EApYYPPA | 6.99 | 7.78 | 1.31 |
| 648 | GSpYKKWC | 2.02 | 43.46 | 0.81 |
| 649 | AApYRAQP | 1.71 | 102.96 | 0.68 |
| 650 | RFpYAYNP | 1.46 | 32.74 | 1.03 |
| 651 | AIpYSGYI | 2.4 | 11.18 | 1.29 |
| 652 | DCpYSRGQ | 2.31 | 4.84 | 0.92 |
| 653 | GApYRGCT | 2.48 | 19.73 | 0.86 |
| 654 | EDpYFPEF | 2.52 | 1.83 | 1.22 |
| 655 | ASpYIDGF | 21.48 | 47.54 | 3.72 |
| 656 | EEpYGYEC | 4.15 | 77.34 | 2.3 |
| 657 | SNpYVFCC | 2.85 | 11.76 | 1.89 |
| 658 | ETpYQPFQ | 2.33 | 15.79 | 1.59 |
| 659 | DTpYKANS | 1.32 | 18.88 | 2.09 |
| 660 | ARpYNGFN | 0.97 | 86.81 | 1.45 |
| 661 | FIpYKPQS | 0.65 | 19.14 | 1.16 |
| 662 | EApYGLDK | 0.71 | 95.32 | 0.88 |
| 663 | DVpYVSYD | 1.31 | 58.26 | 0.84 |
| 664 | LApYMKKN | 1.16 | 0.45 | 0.78 |
| 665 | PVpYCTKT | 2.55 | 51.7 | 0.74 |
| 666 | NLpYYWDQ | 1.17 | 61.7 | 0.64 |
| 667 | DDpYSRDD | 0.58 | 14.89 | 0.56 |
| 668 | ATpYGERV | 0.91 | 109.65 | 0.85 |
| 669 | ATpYGQFY | 0.44 | 0 | 0.45 |
| 670 | AIpYGRNG | 0.95 | 15.93 | 0.41 |
| 671 | EEpYYENV | 0.32 | 7 | 0.58 |
| 672 | ATpYYITK | 0 | 27.8 | 0.11 |
| 673 | ACpYAIHT | 0 | 0 | 0.14 |
| 674 | FDpYQFGY | 0 | 114.14 | 0.86 |
| 675 | QPpYFIPI | 0.35 | 8.13 | 0.76 |
| 676 | DNpYRMKS | 0 | 0 | 0.87 |
| 677 | LEpYCYNP | 0 | 0 | 0.83 |
| 678 | DEpYYRMK | 0 | 0 | 0.76 |
| 679 | GGpYCGYL | 0.59 | 88.46 | 1.43 |
| 680 | EIpYCKGC | 0 | 0 | 1.09 |
| 681 | AApYYGQT | 0 | 0 | 0.95 |
| 682 | NEpYFYVD | 0.1 | 40.03 | 1.04 |
| 683 | NPpYYYLK | 0 | 0.74 | 1.02 |
| 684 | IEpYYLEE | 0.31 | 49.02 | 0.88 |
| 685 | NGpYNDGY | 0.16 | 0 | 0.46 |
| 686 | AIpYSSFK | 0 | 40.35 | 0.97 |
| 687 | APpYPGGG | 0.72 | 47.54 | 1.33 |
| 688 | DDpYRGGG | 0.17 | 10.45 | 1.71 |
| 689 | SSpYPEDN | 106.4 | 121.48 | 2.1 |
| 690 | DDpYQLFE | 0.83 | 22.73 | 1.39 |
| 691 | ERpYHEDA | 2.96 | 42.85 | 1.52 |
| 692 | ASpYYQGL | 0.89 | 31.03 | 1.11 |
| 693 | GSpYGRCQ | 3.76 | 17.28 | 1.18 |
| 694 | AApYGTHL | 1.24 | 27.91 | 0.55 |
| 695 | KVpYMDYN | 3.4 | 36.83 | 1.83 |
| 696 | AApYYLND | 18.31 | 93.45 | 1.86 |
| 697 | EGpYFDAA | 3.9 | 26.14 | 1.66 |
| 698 | KIpYYPDF | 3.57 | 11.9 | 0.65 |
| 699 | GNpYCKYY | 5.05 | 6.51 | 2 |
| 700 | TLpYSFFS | 2.46 | 5.09 | 1.17 |
| 701 | DGpYYRGE | 2.49 | 11.71 | 1.92 |
| 702 | AKpYPFKK | 2.29 | 13.16 | 1.87 |
